# 3-Position Biaryl Endochin-like Quinolones with Enhanced Antimalarial Performance

**DOI:** 10.1021/acsinfecdis.4c00140

**Published:** 2024-06-11

**Authors:** Sovitj Pou, Rolf W. Winter, Rozalia A. Dodean, Katherine Liebman, Yuexin Li, Michael W. Mather, Binod Nepal, Aaron Nilsen, Mason J. Handford, Teresa M. Riscoe, Sydney Laxson, Payton J. Kirtley, Maya Aleshnick, Lev N. Zakharov, Jane X. Kelly, Martin J. Smilkstein, Brandon K. Wilder, Sandhya Kortagere, Akhil B. Vaidya, P. Holland Alday, J. Stone Doggett, Michael K. Riscoe

**Affiliations:** † VA Portland Healthcare System, 3710 SW US Veterans Hospital Road, Portland, Oregon 97239, United States; ‡ Department of Microbiology and Immunology, Drexel University College of Medicine, 2900 Queen Lane, Philadelphia, Pennsylvania 19129, United States; § Department of Chemical Physiology and Biochemistry, Oregon Health & Science University, 3181 SW Sam Jackson Park Road, Portland, Oregon 97239, United States; ∥ Department of Microbiology and Molecular Immunology, Oregon Health & Science University, 3181 SW Sam Jackson Park Road, Portland, Oregon 97239, United States; ⊥ Vaccine & Gene Therapy Institute (VGTI), Oregon Health and Science University (West Campus), 505 NW 185th Avenue, #1, Beaverton, Oregon 97006, United States; # School of Medicine Division of Infectious Diseases, Oregon Health & Science University, 3181 SW Sam Jackson Park Road, Portland, Oregon 97239, United States; △ Center for Advanced Materials Characterization in Oregon (CAMCOR), 1443 E. 13th Avenue, Eugene, Oregon 97403, United States

**Keywords:** antimalarial drug, oral prophylaxis, next-generation ELQs, structure−activity relationships

## Abstract

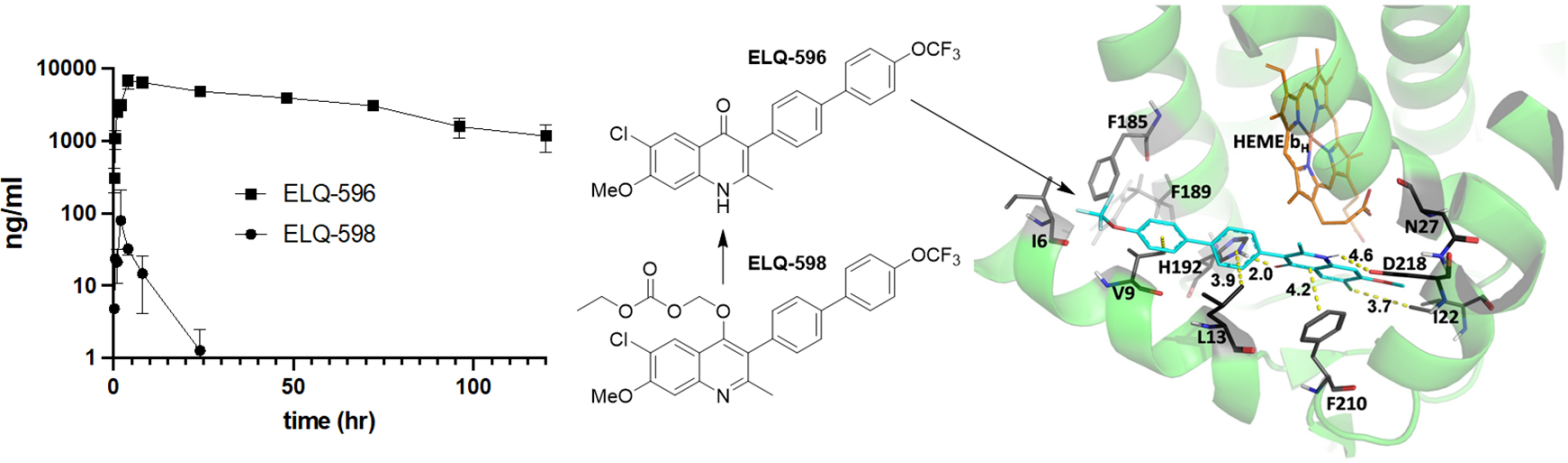

ELQ-300 is a potent antimalarial drug with activity against blood, liver, and vector stages of the disease. A prodrug, **ELQ-331**, exhibits reduced crystallinity and improved in vivo efficacy in preclinical testing, and currently, it is in the developmental pipeline for once-a-week dosing for oral prophylaxis against malaria. Because of the high cost of developing a new drug for human use and the high risk of drug failure, it is prudent to have a back-up plan in place. Here we describe **ELQ-596**, a member of a new subseries of 3-biaryl-ELQs, with enhanced potency in vitro against multidrug-resistant *Plasmodium falciparum* parasites. **ELQ-598**, a prodrug of **ELQ-596** with diminished crystallinity, is more effective vs murine malaria than its progenitor **ELQ-331** by 4- to 10-fold, suggesting that correspondingly lower doses could be used to protect and cure humans of malaria. With a longer bloodstream half-life in mice compared to its progenitor, **ELQ-596** highlights a novel series of next-generation ELQs with the potential for once-monthly dosing for protection against malaria infection. Advances in the preparation of 3-biaryl-ELQs are presented along with preliminary results from experiments to explore key structure–activity relationships for drug potency, selectivity, pharmacokinetics, and safety.

In 2022, an estimated 249 million cases of malaria occurred worldwide with roughly 94% of cases occurring on the African continent. In the same year, there were an estimated 608,000 deaths from malaria around the globe, with children accounting for roughly 76% of all malaria deaths worldwide. Figures from the last 2 years represent an increase in case numbers and deaths over previous years likely because of disruptions in health care delivery due to the COVID pandemic.^1^ Before the pandemic and over the past two decades, the World Health Organization noted steady reductions in cases and deaths worldwide primarily due to an increase in vector control measures and use of mosquito bed nets, as well as the introduction of artemisinin-combined-therapies (ACTs). Now the situation is complicated not only by the continuing effects of the COVID-19 pandemic but also by resistance emerging to ACTs in Asia^2^ and Africa^3^ where resistance to frontline antimalarials such as chloroquine, mefloquine, amodiaquine, antifolates, and quinine is already firmly established. Thus, even though the trend for malaria deaths has generally been on the decline, there is a pressing need for new drugs to address multidrug resistance and to service global efforts toward disease eradication.

To tackle the challenge of today’s dynamic antimalarial drug resistance landscape and to make advances on the goal of worldwide eradication of the disease, the Medicines for Malaria Venture (MMV) has created a list of desirable Target Product Profiles (TPPs) and associated Target Candidate Profiles (TCPs) that provide valuable guidance (or “roadmaps”) for what is needed to achieve the ultimate goal of eradication.^4,5^ The list is comprehensive and includes new oral medications that can be used for treatment of acute but uncomplicated malaria, as well as for severe and complicated disease where a fast-acting parenteral formulation would be appropriate. There is also a TPP for drugs that can be used for prophylaxis where the drug would be given to subjects moving into regions of high malaria endemicity or during epidemics or to especially vulnerable populations, e.g., pregnant women, children, and the elderly. And within these TPPs, there are described drug molecules with TCPs to fill particular niches within the treatment and/or prophylaxis pharmacopoeia of new and available drugs. Such TCPs include drugs that clear asexual blood-stage parasites (TCP-1) or molecules that target the latent liver-stage hypnozoites of *vivax* and *ovale* (TCP-3) or replicating liver schizonts of all malaria species (TCP-4), as well as drugs that interfere with transmission in blood or within the insect vector (TCP-5). More recently, MMV described a new TPP for a long-acting injectable (LAI-C) to be used in treatment and chemoprevention for 2 to 4 months of protection against seasonal malaria or in the case of malaria epidemics.^5^

Over 10 years ago, **ELQ-300** (Figure 1) was discovered as part of a research consortium with the MMV to optimize the historical lead endochin for human use.^6^ Like many cytochrome *bc*_1_ inhibitors, **ELQ-300** is an analog of coenzyme Q, a native ligand of electron transport chain enzymes. [In *Plasmodium* species, the number of isoprenyl groups in the CoQ side chain is 8 to 9.^7^] Since its discovery, nearly everything that we have learned about **ELQ-300** shows that it would be a highly valuable addition to the antimalarial toolbox for the prevention and treatment of malaria and for transmission blocking.^6^ The distinguishing characteristics of the drug include the following: low nM IC_50_’s vs multidrug-resistant strains of *P. falciparum* including field isolates; pan-antimalarial activity against the various *Plasmodium* species that infect humans;^8^ potent activity against replicating parasites in the liver,^6^ blood, and vector stages of infection;^6^ novel and selective targeting of the Q_i_ site of *P. falciparum* cytochrome *bc*_1_ complex;^9^ excellent metabolic stability and extended pharmacokinetics in preclinical species (mouse, rat, and dog); and a clean safety profile.^6^ Although this was sufficient for **ELQ-300** to be selected as a preclinical candidate by the MMV in 2012, further development was derailed in 2014 when it was dropped from the pipeline due to high crystallinity that limited absorption and prevented determination of an in vivo therapeutic index necessary for regulatory approval. Fortunately, we were able to address this issue and revive interest in **ELQ-300** by introduction of a prodrug (**ELQ-331**, Figure 1) with significantly reduced crystallinity that gave improved oral bioavailability and enhanced overall antimalarial performance.^10^**ELQ-331** was accepted as a preclinical candidate by the MMV in October of 2020. Since this time, an oral formulation of **ELQ-331** has been developed by the MMV, and we recently described a low-cost and scalable synthetic route to the core molecule **ELQ-300** adding to the feasibility of developing this drug for human use.^11^ Thus, prodrug **ELQ-331** continues to move forward through the MMV clinical development pipeline.

**Figure 1:**
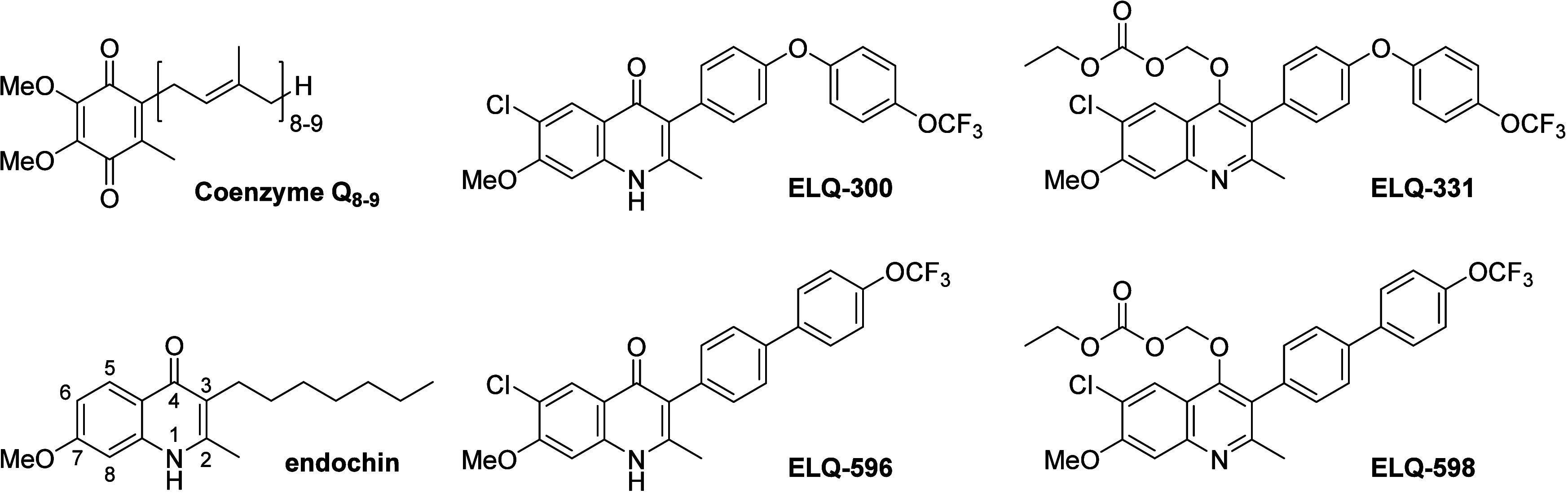
Structures of coenzyme Q_8–9_, endochin, **ELQ-300**, **ELQ-331**, **ELQ-596**, and **ELQ-598**.

In drug development, it is important to plan both for success and failure. In a 2016 report published in the *Journal of Health Economics*, scientists from the Tufts Center for the Study of Drug Development estimated the cost of developing a new drug for human use at roughly $2.6 billion dollars.^12^ Added to this staggering cost is the fact that only ∼12% of drugs entering clinical development actually gain regulatory approval. For this reason, it is prudent to have a back-up plan, and so we continue to explore the structure–activity relationships of ELQs in search of improvements in intrinsic potency, selectivity, pharmacokinetic properties, and/or efficacy. Here we describe a close structural analog of **ELQ-300**, **ELQ-596**, in which the 3-position diphenylether is replaced by a biphenyl group. This molecule exhibits improved potency against multidrug-resistant *P. falciparum* strains including a clinical isolate harboring resistance to atovaquone. In vivo efficacy in a murine model of malaria is also enhanced over its progenitor. Taken together, our discovery highlights a new series of substituted 3-biaryl-ELQs that represent backups should **ELQ-331** fail to progress during future clinical investigations or next-generation ELQs with enhanced profiles in potency, efficacy, and pharmacokinetics.

## Chemistry

**Scheme 1:**
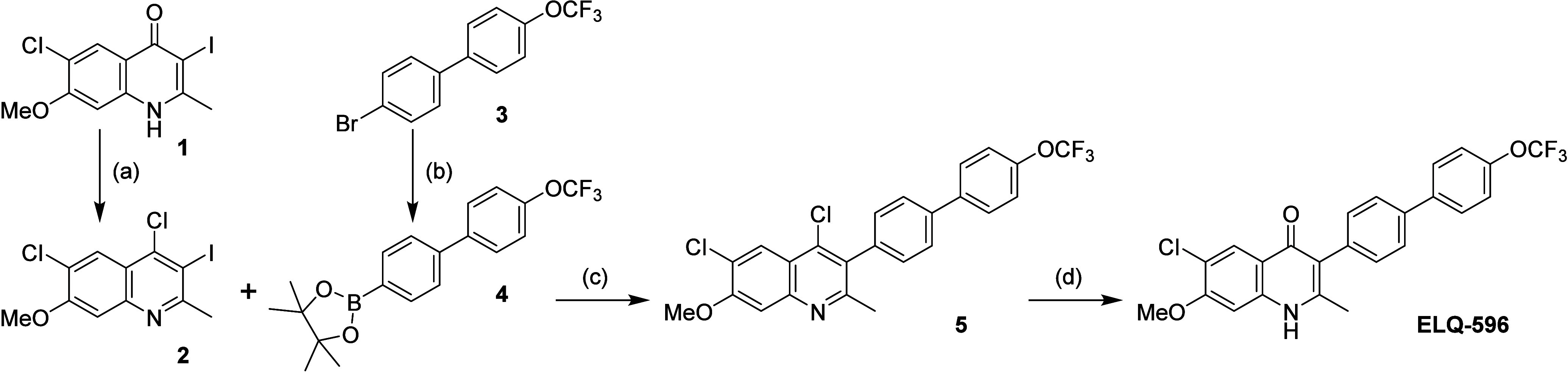
Synthesis of **ELQ-596** Reaction (a): POCl_3_, methylene chloride (DCM), reflux, 93%; (b): Pd(dppf)Cl_2_, bis(pinacolato)diboron, KOAc, DMF, 80 °C, 71%; (c): Pd(dppf)Cl_2_, K_2_CO_3_, DMF, 80 °C, 71%; (d): KOAc, AcOH, 120 °C, 85%.

Initially, **ELQ-596** was prepared using a previously reported approach^13^ with one important change. The 4(1*H*)-quinolone **1** was protected as a 4-chloro-quinoline (**2**) using phosphorus oxychloride^14^ (POCl_3_) (Scheme 1) rather than as a 4-*O*-ethyl-quinoline. The pinacol ester **4** was prepared from commercially available 4-bromo-4′-(trifluoromethoxy)-1,1′-biphenyl **3** and selectively coupled^15^ to the quinoline **2** as previously described.^13^ Finally, the 4(1*H*)-quinolone **ELQ-596** was obtained after hydrolysis of the 4-chloro-quinoline **5** using potassium acetate (KOAc) in glacial acetic acid (AcOH). This method worked well for the preparation of **ELQ-596** but was not useful for structural variation because of the lack of availability and relative expense of substituted 4-bromo-1,1′-biphenyl starting materials.

**Scheme 2:**
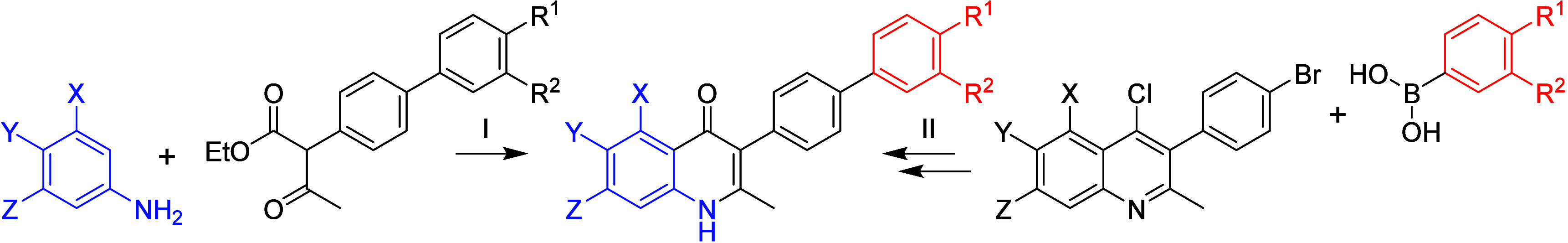
Two Different Approaches to the Chemical Synthesis of 3-Biaryl-ELQs Substituted in the Quinolone Core or Biaryl Side Chain

With **ELQ-596** in hand, it was desirable to vary the structure to determine if inhibitory activity and solubility could be improved. To vary the benzenoid substituents X, Y, and Z, we decided to adopt approach I (Scheme 2), where the 4(1*H*)-quinolone is formed in the final reaction step. To vary the biphenyl substituents R^1^ and R^2^, approach II was used wherein the outer ring of the biphenyl side chain was introduced late in the 4(1*H*)-quinolone synthesis.

**Scheme 3:**
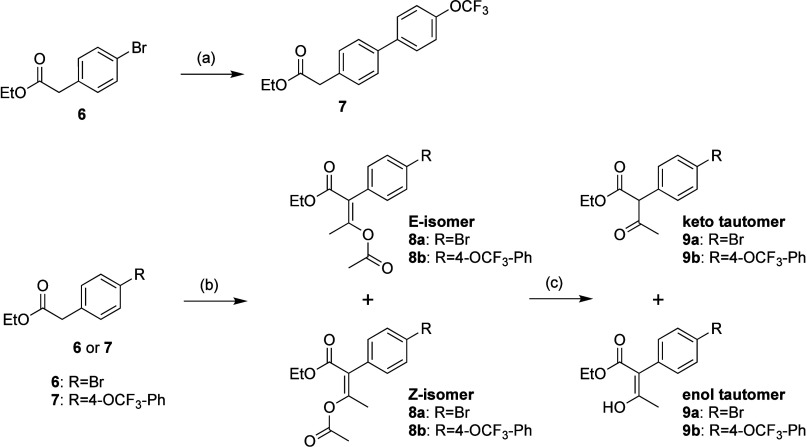
Synthesis of β-Keto Esters **9a** and **9b** Reaction (a): (4-(trifluoromethoxy)phenyl) boronic acid, Pd(dppf)Cl_2_, K_2_CO_3_, DMF, 80 °C, 58%; (b): LiHMDS, Ac_2_O, and THF, −20 °C to RT over 20–72 h; (c) 10% *p*-TsOH and AcOH, 100 °C over 2–16 h, 89–100%.

To prepare the β-keto ester intermediates **9a** and **9b**, we adapted a route that we recently developed for the large-scale synthesis of **ELQ-300.**(11) Suzuki reaction of commercially available ethyl 2-(4-bromophenyl) acetate **6** and (4-(trifluoromethoxy)phenyl) boronic acid with [1,1′-bis(diphenylphosphino)-ferrocene]palladium(II) dichloride (Pd(dppf)Cl_2_) and potassium carbonate (K_2_CO_3_) in dimethylformamide (DMF) provided ethyl 2-(4′-(trifluoromethoxy)-[1,1′-biphenyl]-4-yl)acetate **7** in 58% yield (Scheme 3). Acylation of **6** and **7** using freshly prepared lithium hexamethyldisilazide (LiHMDS) and excess acetic anhydride (Ac_2_O) in tetrahydrofuran (THF) gave bis-acylated enol acetates **8a** and **8b** as mixtures of equally reactive E- and Z-isomers in quantitative yield and in sufficient purity (>95%) to be used in the next step without further purification. The two stereoisomers can be isolated by flash chromatography. However, we were not able to unambiguously assign the two stereoisomers using NOESY 2D NMR. GC–MS analysis indicated that the major stereoisomer of **8a** was 80% of the mixture, whereas the major stereoisomer of **8b** was 95% of the mixture. Using catalytic *para*-toluenesulfonic acid (*p*-TsOH) in AcOH, bis-acylated **8a** and **8b** were converted to β-keto ester intermediates **9a** and **9b**, which existed as mixtures of keto and enol tautomers as determined by ^1^H NMR. After concentration, the crude reaction mixture contained mainly β-keto esters **9a** or **9b** and catalytic *p*-TsOH (required in the next reaction) and was usable without further purification.

**Scheme 4:**
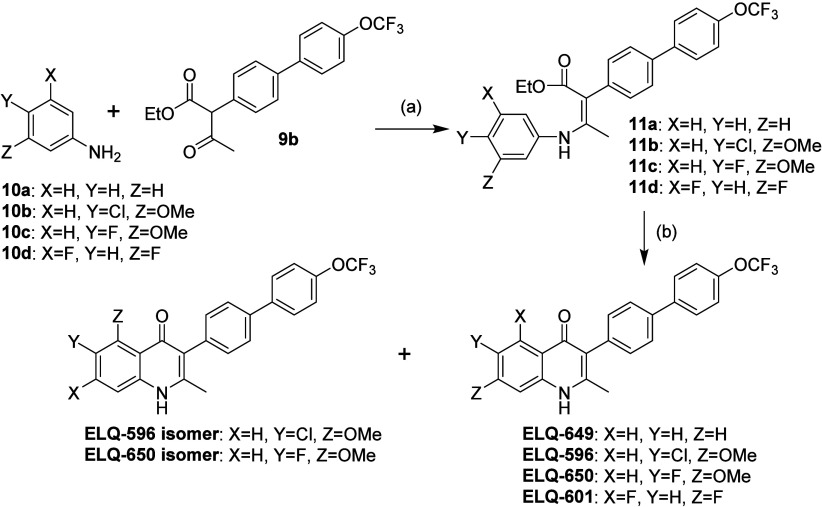
Synthesis of a Series of 3-Biaryl-ELQs with Variable Substitution of the Benzenoid Ring (X, Y, and Z) from β-Ketoester **9b** Reaction (a): 10% *p*-TsOH, benzene, reflux, 24–72 h; (b): Dowtherm A, 250 °C, 5 min, 28–37%.

Relatively facile variation of benzenoid substituents X, Y, and Z was accomplished by reaction of β-keto ester **9b** with various anilines (**10a**–**d**). The crude β-keto ester mixture was reacted with anilines **10a**–**d** under Dean–Stark conditions with refluxing benzene to provide Schiff bases **11a**–**d**, which were used without further purification (Scheme 4). Formation of 4(1*H*)-quinolones **ELQ-596**, **ELQ-601**, **ELQ-649**, and **ELQ-650** was accomplished via Conrad–Limpach cyclization^16^ of Schiff bases **11a**–**d** in Dowtherm A at 250 °C.^17^ The crude products obtained from this reaction were >98% pure as determined by HPLC and ^1^H NMR. To allow detection and quantification of the **ELQ-596** and **ELQ-650** regioisomers, the products were converted to their corresponding 4-chloro derivatives using POCl_3_ and analyzed by GC–MS. The results indicated that negligible amounts (<0.5%) of **ELQ-596 isomer** and **ELQ-650 isomer** were formed under these conditions.

**Scheme 5:**
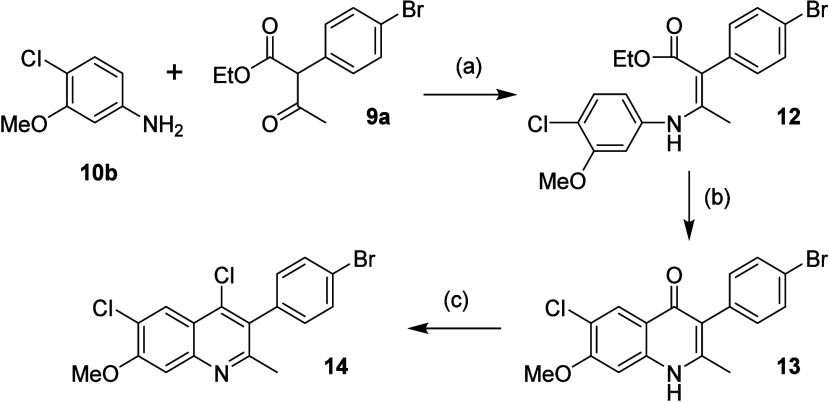
Synthesis of 3-(4-Bromophenyl)-4,6-dichloro-7-methoxy-2-methylquinoline **14** from β-Ketoester **9a** Reaction (a): 10% *p*-TsOH, benzene, reflux, 21 h; (b): Dowtherm A, 250 °C, 5 min, 50%; POCl_3_, reflux, 45 min, 100%.

Synthesis of 4(1*H*)-quinolone **13** was accomplished using the same method described above. The crude β-keto ester **9a** mixture was reacted with commercially available 4-chloro-3-methoxy aniline **10b** under Dean–Stark conditions with refluxing benzene to provide Schiff base **12**, which was used without further purification (Scheme 5). Formation of 4(1*H*)-quinolone **13** was accomplished via Conrad–Limpach cyclization^16^ of Schiff base **12** in Dowtherm A at 250 °C.^17^ 4-Chloroquinoline **14** was then prepared from 4(1*H*)-quinolone **13** using neat POCl_3._^14^

**Scheme 6:**
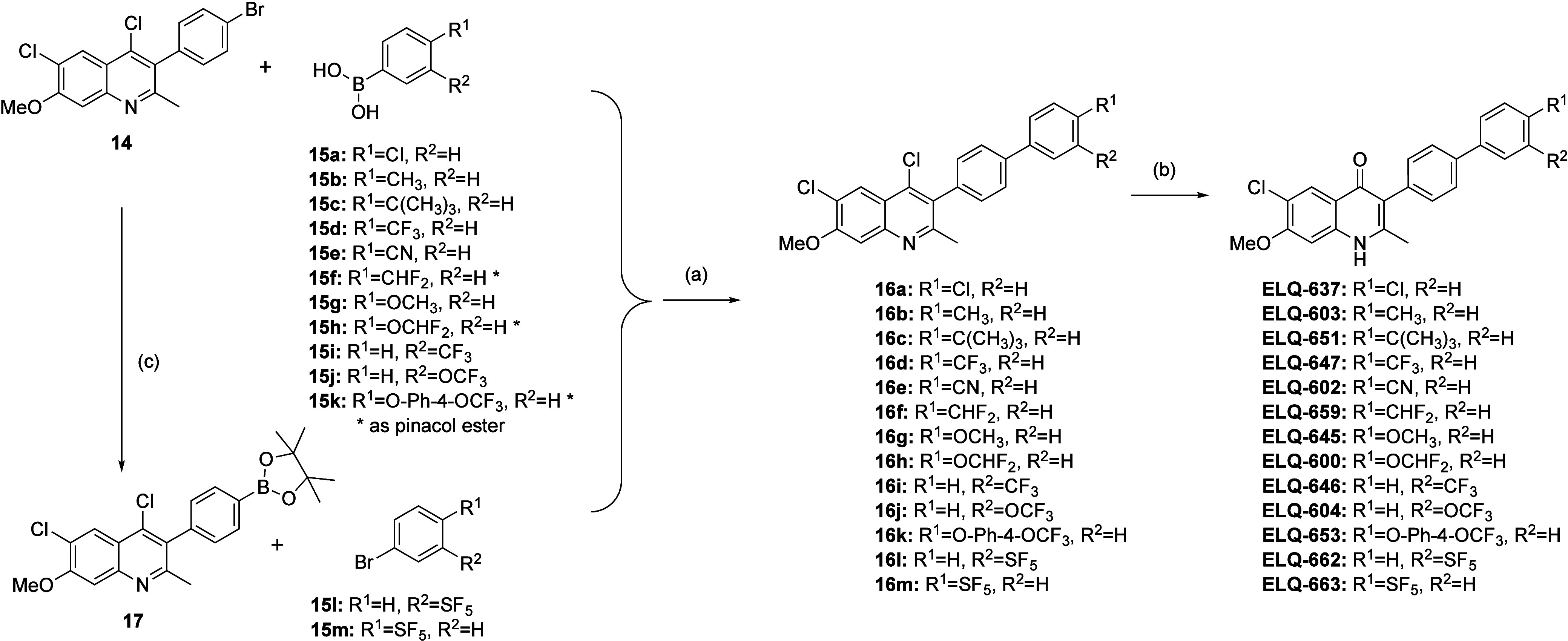
Synthesis of a Series of 3-Biaryl-ELQs with Structural Variation at the Terminal Benzene Ring Reaction (a): Pd(dppf)Cl_2_, K_2_CO_3_, DMF, 80 °C, 11–67%; (b): KOAc, AcOH, 16–24 h, 36–87%, (c): Pd(dppf)Cl_2_, BPin_2_, KOAc, DMF, 80 °C, 46%.

Variation of biphenyl substituents R^1^ and R^2^ was then accomplished via selective Suzuki reaction of various boronic acids or pinacol esters **15a**–**k** with 4-chloro-quinoline **14** using Pd(dppf)Cl_2_ and K_2_CO_3_ in DMF (Scheme 6). For compounds **16l** and **16m**, compound **14** was converted to a boronic ester by a method analogous to that shown for compound **4** (Scheme 3); this boronic ester was allowed to react with the appropriate bromophenyl sulfur pentafluoride compound using Pd(dppf)Cl_2_ and K_2_CO_3_ in DMF. The resulting 4-chloro-quinolines **16a**–**m** were then converted to their corresponding 4(1*H*)-quinolones using KOAc in AcOH (Scheme 6).

**Scheme 7:**
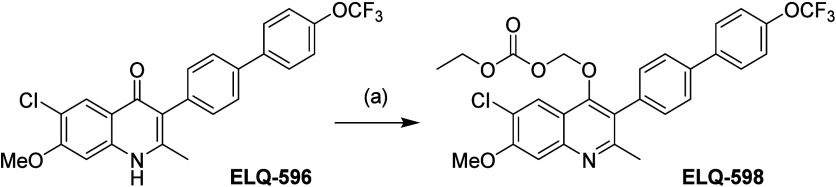
Synthesis of Alkoxy Carbonate Prodrug **ELQ-598** Reaction: (a) chloromethyl ethyl carbonate, TBAI, K_2_CO_3_, and DMF, 60 °C, 24 h, 73%.

For our in vivo work, it was necessary to convert **ELQ-596** to the corresponding alkoxycarbonate ester prodrug **ELQ-598.** This was accomplished using chloromethyl ethyl carbonate, tetra-*n*-butylammonium iodide (TBAI), and K_2_CO_3_ in DMF according to a previously published method (Scheme 7).^10^

## Results and Discussion

### Rationale for the Synthesis of a 3-Biaryl Version of ELQ-300

We maintain a large chemical library of >500 ELQ derivatives, and over the years, we and others have evaluated these drugs for antiparasitic activity against a range of parasites including *Plasmodium falciparum*, *Toxoplasma gondii*, *Babesia microti*, and *B. duncani*. All of this information is stored in a database with information relating to chemical structure, physical chemical properties, cross resistance patterns, mammalian cell cytotoxicity, enzyme inhibitor profiles, and synthesis details. Pharmacological data and in vivo efficacy data are also stored in the database for molecules of particular interest. Recently, we conducted a retrospective analysis of the compounds that we have made over the years and came to the realization that neither we nor anyone else had ever synthesized the biphenyl analog of **ELQ-300**, i.e., **ELQ-596** (Figure 1). We synthesized the compound in an initial batch size of about 800 mg as described above. **ELQ-596** was then tested for antiplasmodial activity vs four lab strains of *P. falciparum* including the drug-sensitive D6 strain, the multidrug-resistant Dd2 strain, the atovaquone (ATV)-resistant clinical isolate Tm90-C2B,^18^ and the **ELQ-300**-resistant D1 clone,^9^ previously isolated from Dd2. In vitro assays were conducted in quadruplicate in 96-well plates in a starting range of 250 to 0.25 nM and retested at a lower range when drug potency extended below this initial range, i.e., 62.5 to 0.06 nM. Twofold dilutions were made across the tested range to determine IC_50_ values. We employed the SyBr Green assay, and the plates were incubated for 72 h before harvesting.^19,20^ Fluorescence readings were captured with a fluorescence plate reader and processed by nonlinear regression analysis using the GraphPad Prism software to provide IC_50_ values for each determination. Stock solutions were freshly prepared daily in DMSO in glass vials.

### In Vitro Activities of Selected 3-Biaryl-ELQs vs *P. falciparum* Strains

Mean IC_50_ values are shown in Tables 1 and 2 for experiments performed in quadruplicate. We also prepared **ELQ-598**, the alkoxy-carbonate ester prodrug of **ELQ-596**, and included it in the assays along with historical controls atovaquone (ATV), **ELQ-300**, and **ELQ-400**. The latter is a drug that, like ATV, targets the Q_o_ site of the *Pf* cyt *bc*_1_ complex.^21^ Notice that the in vitro IC_50_ values for **ELQ-596** were improved over **ELQ-300** by 5- to 7-fold for the D6 and Dd2 strains as well as the ATV^r^ C2B (Table 1), whereas they were higher for the **ELQ-300**^**r**^ D1 clone. We interpret these results to suggest higher inhibitory action by **ELQ-596** vs the wild-type *Pf* cyt *bc*_1_ as well as the mutated cyt *bc*_1_ complex of the clinical isolate Tm90-C2B.

**Table 1:**
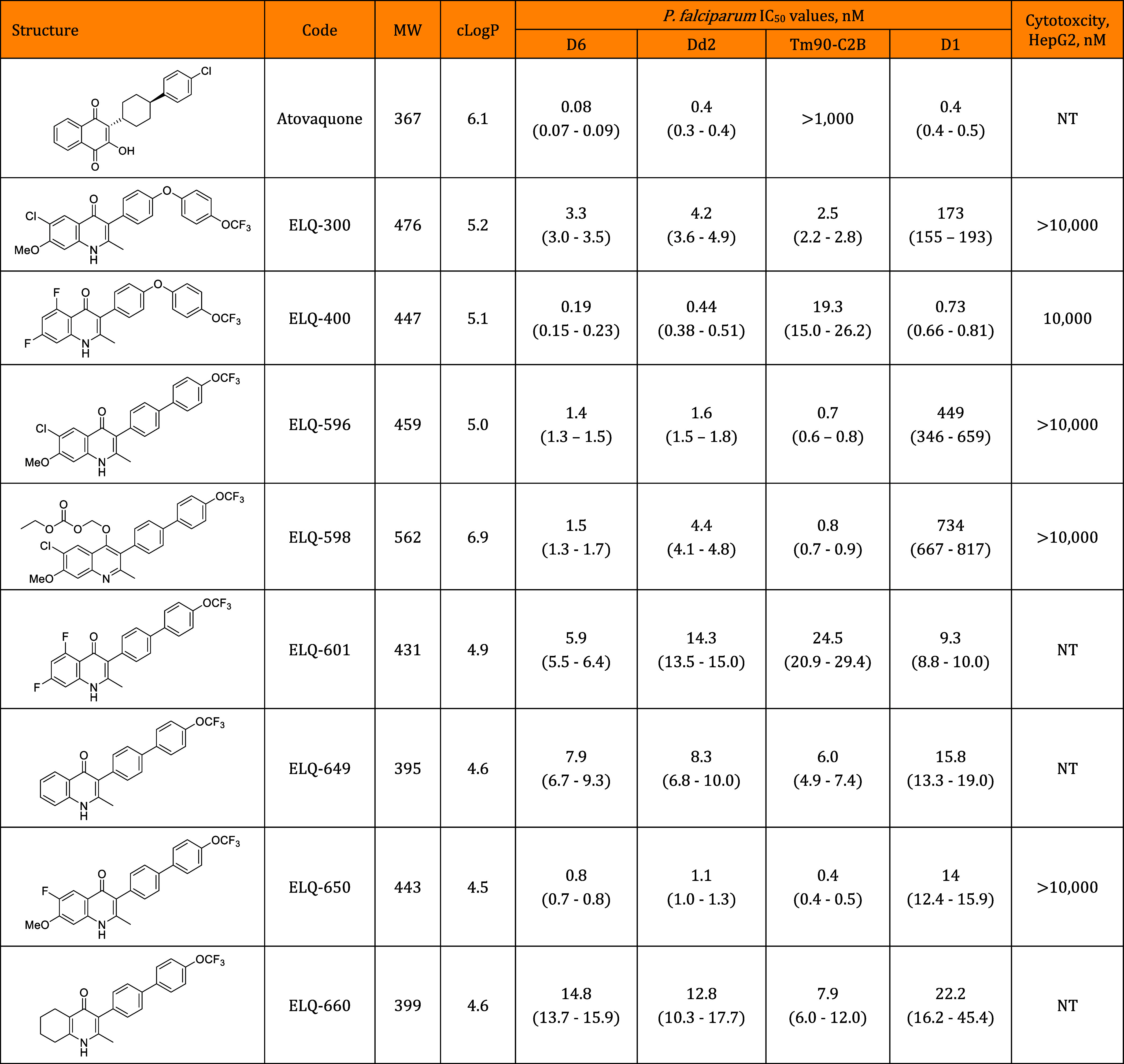
Structure Activity Profile of 3-Biaryl-ELQs vs. Drug-Sensitive (D6) and Drug-Resistant (Dd2, C2B, and D1) Strains of *Plasmodium falciparum*: Impact of Substitutions to the Quinolone Core^a^

a IC_50_ values represent the concentration of drug that suppresses parasite growth by 50% relative to controls without addition of drug. For mean IC_50_ values, minimally quadruplicate data were collected for each concentration in the dose–response curves, and error represents the 95% confidence interval of the fit. For each test agent, we show the results from a single representative experiment. Cytotoxicity was assessed in HepG2 cells in a galactose containing medium to reverse the Crabtree effect and force the cells to rely on their mitochondria to produce ATP. Cytotoxicity assessment utilized the Titerglo II reagent as detailed in the Materials and Methods section. The positive control rotenone exhibited an IC_50_ of <1000 nM. Calculated cLog*P* values are from ChemDraw version 20.1.

**Table 2:**
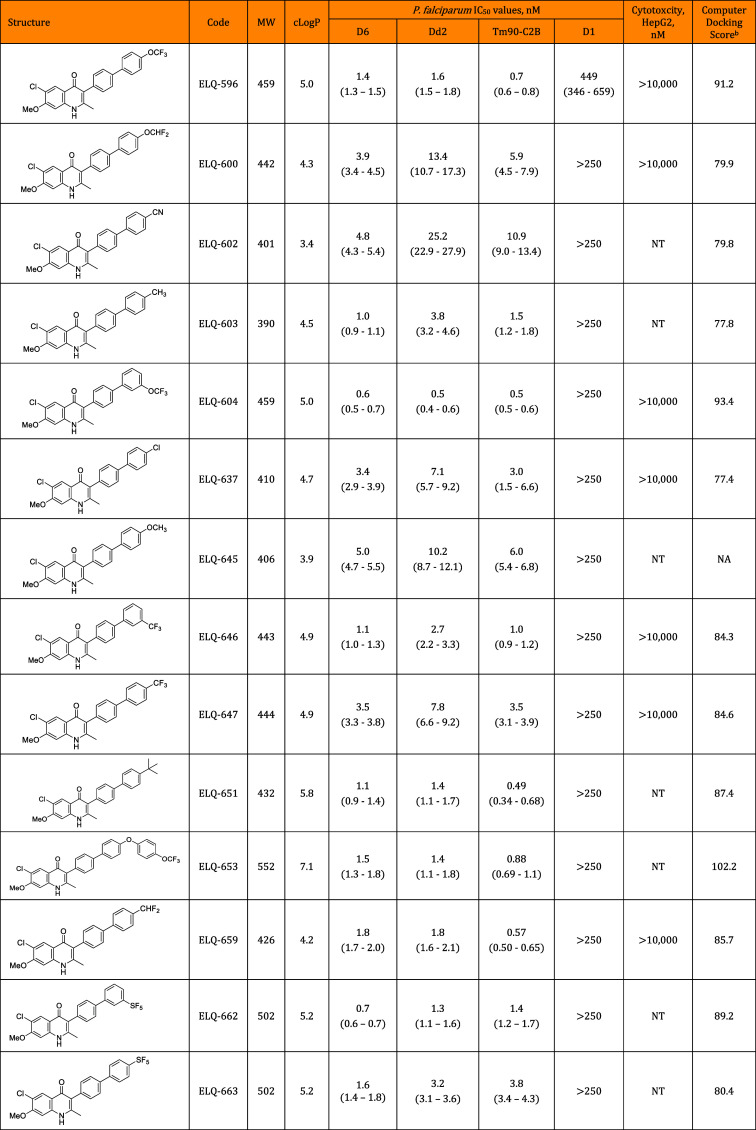
Structure Activity Profile of 3-Biaryl-ELQs vs. Drug-Sensitive (D6) and Drug-Resistant (Dd2, C2B, and D1) Strains of *P. falciparum*: Impact of Outer Ring Substitutions^a^

a IC_50_ values represent the concentration of drug that suppresses parasite growth by 50% relative to controls without addition of drug. For mean IC_50_ values, minimally quadruplicate data were collected for each concentration in the dose–response curves, and error represents the 95% confidence interval of the fit. For each test agent, we show the results from a single representative experiment. Cytotoxicity was assessed in HepG2 cells in galactose containing medium to reverse the Crabtree effect and force the cells to rely on their mitochondria to produce ATP. Cytotoxicity assessment utilized the Titerglo II reagent as detailed in the Materials and Methods section. Calculated cLog*P* values are from ChemDraw version 20.1.

b GOLD docking scores for selected ELQs and their correspondence to antiplasmodial IC_50_ values vs D6 and Dd2 strains.

### ELQ-596 Metabolic Stability

We then evaluated **ELQ-596** for metabolic stability in the presence of pooled murine hepatic derived microsomes. Because of its close structural similarity to **ELQ-300**, we expected the new analog to be stable under the conditions of the assay. The drug was incubated in the presence of pooled murine liver microsomes (0.5 mg/mL) at 37 °C in the presence of NADPH to test for P450 drug dependent metabolism. Samples were taken over the interval of 45 min and analyzed by LC–MS/MS for the presence of test compound. Ketanserin served as an internal standard for the metabolic rate of a drug with known intermediate stability. As shown in Table 3, tests demonstrated extreme stability of **ELQ-596** to microsomal attack with negligible breakdown over the course of 45 min of incubation, yielding an estimated *T*_1/2_ in this in vitro system of >4000 min. The microsomal stability of **ELQ-596** extended to other species as well including rats and humans.

**Table 3:** Metabolic Stability of **ELQ-596** in the Presence of Liver Microsomes

test compound	species	*T*_1/2_ (min)	Cl_int_ (mL/min/kg)
ketanserin	mouse	19.10	285.69
	rat	14.41	172.38
	human	27.29	63.69
**ELQ-596**	mouse	>4000	0
	rat	>4000	0
	human	>4000	0

### In Vivo Efficacy of **ELQ-596** and Alkoxycarbonate Ester Prodrug **ELQ-598** against Murine Malaria

Next, we were interested in testing **ELQ-596** in vivo. Because it is a highly crystalline compound like **ELQ-300**, we prepared an alkoxycarbonate ester prodrug, **ELQ-598**. And like **ELQ-331**, we found that **ELQ-598** exhibits significantly reduced crystal lattice strength as evidenced by a 229 °C decrease in melting point (Table 4). [We include a comparison of the X-ray crystal structures of **ELQ-598** and **ELQ-331** in the Supporting Information, Figures S1–S4.] We tested **ELQ-598** in a 4 day test using a modified Peters protocol in which all test animals are first inoculated with 35,000 infected red cells from a donor mouse infected with *P. yoelii* via tail vein injection (day 0). Animals were then dosed with **ELQ-598** dissolved in PEG400 (100 μL) by oral gavage on days 1, 2, 3, and 4. On day 5, a drop of blood was taken from the tail, and a blood smear was prepared, fixed with methanol, and stained with Giemsa. The operator then examined the stained smear microscopically to determine percent parasitemia. (Typically, control animals exhibit a level of parasitemia on day 5 of ∼20%.) Dosages of 0.0025, 0.005, 0.01, 0.03, 0.1, 0.3, 1.0, and 10 mg/kg/day were used for the initial experiment. From two separate studies (four mice per group), the average estimates for ED_50_ and ED_90_ along a very sharp action curve were 0.005 and 0.0065 mg/kg/day, respectively, with a nonrecrudescence dose (NRD) of 0.12 mg/kg/day (Table 4). These values are 4- to 8-fold lower than for **ELQ-300** and **ELQ-331**. Gratifyingly, the superiority of **ELQ-596** carried over to single dose cures (SDCs) for prodrug **ELQ-598**. In this model, animals were inoculated exactly as for the 4 day test on day 0; however, drug was administered only on day 1, whereas smears were made on day 5 and again weekly thereafter for animals that remained aparasitemic. Animals that remained aparasitemic on day 30 were scored as cures. In this latter experiment, the lowest fully protective single dose cure was at 0.3 mg/kg (0.34 mg/kg of prodrug). The lower dose of 0.25 mg/kg **ELQ-598** protected two of four animals. Thus, prodrug **ELQ-598** is ∼10 times more effective as a single dose cure agent against blood-stage *P. yoelii* infections in mice when compared directly to **ELQ-331** (the lowest SDC in this model was 3 mg/kg).

**Table 4:** Comparison of **ELQ-300**, Prodrug **ELQ-331**, and **ELQ-596** and Prodrug **ELQ-598**^a^

code	melting point, °C	blood stage in vivo efficacy, mg/kg/day				liver stage in vivo	
		4 day Peters test results			single dose cure	dose mg/kg/day	# mice protected/total
		ED_50_	ED_90_	NRD			
**ELQ-300**	316	0.02	0.06	1.0	>25	NT	NT
**ELQ-331**	117	0.02	0.05	1.0	3	1.0 (single dose)^b^	4/4
**ELQ-596**	365	0.01	0.021	0.3	NT	NT	NT
**ELQ-598**	136	0.005	0.0065	0.12	0.3	1.0 (single dose)^b^	4/4
ATV	217	0.1	ND	10	10	NT	NT
CQ	NT	1.6	2.7	>64	>64	NT	NT

a MP = melting point; ED_50_ = dose required to suppress parasitemia by 50% relative to untreated controls (4 day Peters test), ED_90_ = dose required to suppress parasitemia by 90% relative to untreated controls (4 day Peters test, *P. yoelii* Kenya Strain), NRD = nonrecrudescence dose (4 day Peters test), SDC = single dose cure (lowest single dose that provides complete cures of all four mice in the group), NT = not tested, ND = not determined. Note: Prodrugs were dosed based on molar equivalency to the parent drug. For liver-stage experiments, the animals received an inoculation of 10,000 *P. yoelii* sporozoites 1 h before drug dosing as detailed in the methods. Protection was assessed on days 3 and 5 and weekly thereafter for 30 days.

b Lowest dose tested under these conditions.

### In Vivo Protective Efficacy of Prodrug **ELQ-598** against Sporozoite Challenge by *P. yoelii* in Mice

We also assessed the causal prophylactic activity of **ELQ-596** in the form of the prodrug **ELQ-598**. For this study, mice were inoculated with 10,000 *P. yoelii* sporozoites via tail vein injections, and drug was administered by gavage an hour later and only once. We monitored for the presence of blood-stage parasites as a measure of liver-stage infection. Blood smears were taken and fixed and stained with Giemsa at 72 and 120 h and weekly thereafter until day 30 before scoring animals as fully protected. As shown in Table 4, **ELQ-598** provided complete protection against sporozoite challenge with a single oral dose of 1.0 mg/kg, the lowest dose attempted to date. The positive control drug **ELQ-331** was also completely protective at this same dose. Importantly, because the pre-erythrocytic stage of *P. yoelii* is relatively short, i.e., only ∼48 h, we consider this activity to be for “presumptive efficacy”, which will need to be confirmed in another system perhaps using bioluminescence whole animal imaging.

### Pharmacokinetics of **ELQ-596** following a Single Dose of Prodrug **ELQ-598**

**Figure 2:**
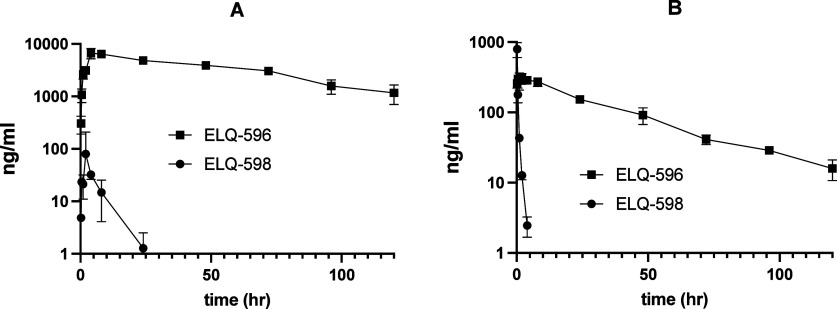
Mean **ELQ-596** and **ELQ-598** plasma concentration–time profiles after dosing with a single dose **ELQ-598**: (A) 10 mg/kg PO dose and (B) 0.3 mg/kg IV dose.

Plasma concentration versus time curves for **ELQ-598** and **ELQ-596** following single dose gavage (PO) or intravenous (IV) administration of **ELQ-598** to mice are shown in Figure 2, and the resulting pharmacokinetic (PK) parameters are summarized below in Table 5. After 0.3 mg/kg IV (an equivalent dose of 0.25 mg/kg **ELQ-596)**, the plasma concentration of **ELQ-598** rapidly fell below the lower level of quantitation (LLOQ), indicating rapid conversion to **ELQ-596**. The plasma concentration of **ELQ-596** reached *C*_max_ (303 ng/mL) 4 h after dosing and showed an elimination *T*_1/2_ = 27.4 h. After 10 mg/kg PO (an equivalent dose of 8.2 mg/kg **ELQ-596**), the concentration of **ELQ-598** peaked within 2 h and again fell rapidly, but it was still detectable 24 h after dosing in PEG400. Plasma **ELQ-596***C*_max_ and *T*_max_ were 6683 ng/mL (14.5 μM) and 4 h, respectively, and the apparent elimination *T*_1/2_ was 45.7 h.

**Table 5:** Disposition Characteristics of **ELQ-596** and **ELQ-598** following a Single **ELQ-598** Dose of 10 mg/kg PO and 0.3 mg/kg IV^a^

PK parameter estimate	ELQ-596		ELQ-598	
	IV	PO	IV	PO
dose (mg/kg)	0.25^b^	8.19^b^	0.30	10.0
*k*_el_ (h^–1^)	0.025	0.015	0.93	0.14
*T*_max_ (h)	2	4	ND	2.0
*C*_max_ (ng/mL)	303	6683	ND	79.4
*T*_1/2_ (h)	27.4	45.7	0.74	5.0
Vd_area_/*F* (L/kg)	0.81	1.11	0.42	173.5
Vd_ss_ (L/kg)	0.78	ND	0.09	ND
CL/*F* (L/h/kg)	0.020	0.017	0.40	23.86
AUC_0-inf_ (ng·h/mL)	12197	484353	757	419
*F* (%)	100	73	100	0.26
AUC_ratio_ (%)			5.1	0.07

a Note: Prodrug **ELQ-598** was dissolved in a vehicle composed of 10% DMSO, 10% Solutol HS 15, and 80% saline to yield clear solutions at 0.06 mg/mL for IV dosing. For PO dosing, **ELQ-598** was dissolved in 100% PEG400 to yield clear solutions at 2 mg/mL prior to dosing. ND, not determined.

b Denotes the effective **ELQ-596** dose as derived from MW assuming the complete conversion of prodrug **ELQ-598** to active metabolite **ELQ-596** in vivo. PK parameters for **ELQ-596** are thus “apparent values”.

Two observations after PO dosing of **ELQ-598** appear discrepant with results after IV dosing and thus require brief comment: the apparent persistence, albeit minimal, of **ELQ-598** and the apparent increased elimination *T*_1/2_ of **ELQ-596**. We believe that neither represents an actual dosing route-dependent change in elimination and that both are the result of sustained and prolonged gastrointestinal (GI) absorption of **ELQ-598** (and possibly **ELQ-596** through bile recycling) in this model. The **ELQ-596** elimination profile after IV dosing reflects the intrinsic *T*_1/2_ for **ELQ-596**, and the ultimate significance of the sustained GI absorption of **ELQ-598** seen here will be assessed in future studies using different oral formulations and other animal species.

Most importantly, in comparison to its predecessor prodrug **ELQ-331** (active metabolite, **ELQ-300**), the results of **ELQ-598** dosing indicate improvement in two critical **ELQ-596** PK parameters favoring feasible long-acting efficacy (high bioavailability [*F*] and extended *T*_1/2_) and confirm rapid and complete conversion from prodrug to active metabolite. Using the assumptions needed to estimate the apparent *F* for an active metabolite of a prodrug, **ELQ-596** shows excellent oral bioavailability (73%) in this model (Table 5). The 27.4 h elimination *T*_1/2_ of **ELQ-596** is substantially longer than that of **ELQ-300** (14.6 h) and is noteworthy. Lastly, as with earlier prodrugs in this series, **ELQ-598** exposure is minimal and transient: the molar ratios of AUC_ELQ-598_/AUC_ELQ-596_ after IV and PO dosing were 5.1 and 0.07%, respectively, consistent with the rapid and complete conversion of the prodrug to active **ELQ-596** by host esterases. Taken together, these findings suggest that **ELQ-598** may have significant advantages as an effective agent to deliver very long-acting antimalarial prophylaxis in diverse applications with oral, injectable, or implantable formulations.

### Lack of Inhibition of Human Cytochrome *bc*_1_ Complex by **ELQ-596**

We had previously found that 6-chloro and 7-methoxy groups on the benzenoid ring of the quinolone core prevented **ELQ-300** from interacting with and inhibiting the human cytochrome *bc*_*1*_ complex at concentrations up to 10 μM or higher. For this reason, we expected that **ELQ-596** would show a similar profile. Accordingly, we evaluated **ELQ-596** for inhibition of the human host cytochrome *bc*_1_ complex isolated from human liver tissue and found no detectable inhibition across a concentration range from 156 to 10,000 nM (Table 6). Antimycin A served as a positive control for these assays, exhibiting an EC_50_ vs human cytochrome *bc*_*1*_ of less than 156 nM under assay conditions described in the Materials and Methods section. Given that bloodstream concentrations of **ELQ-596** are unlikely to ever approach 10 μM in clinical use, these results suggest a low potential for side effects in humans due to inhibition of the host cytochrome *bc*_1_ enzyme complex by this drug.

**Table 6:** Comparative Inhibition of Human (Host) Cytochrome *bc*_1_ Complex

**compound**	**human liver cytochrome***bc*_**1**_**, EC**_**50,**_**nM**
atovaquone	460^a^
**ELQ-300**	>10,000^a^
**ELQ-596**	>10,000

a Data taken from Nilsen et al., 2013. Assay conditions are presented in the Materials and Methods section.

### Safety and Mitochondrial Toxicity of **ELQ-596**

Of course, enhanced antiplasmodial activity is desirable only if unaccompanied by enhanced toxicity. Although our in vivo efficacy-testing model is not intended as a formal toxicity assessment, there were no appearance, behavioral, or weight changes observed after dosing with **ELQ-598** at any dose level. We also evaluated **ELQ-596** for cytotoxicity using the TiterGlo luminescence assay kit, which determines cell viability by measuring cellular ATP. In the assay, ATP is consumed as a cosubstrate of luciferase on reaction with its substrate luciferin with the release of light. Using the human HepG2 cell line in the culture medium in which glucose was replaced by galactose to promote reliance upon oxidative phosphorylation processes and to reverse the so-called “Crabtree effect”, we observed an EC_50_ of >10 μM for **ELQ-596** (Table 7). Under the same conditions, the control drug, rotenone, nearly completely eliminated the ATP signal at all concentrations in the range of 1 to 10 μM (Table 7). The incubation period for these experiments was 24 h. Other selected 3-biaryl-ELQs were evaluated for cytotoxicity in this assay, and the values appear in Tables 1 and 2. In summary, our findings show that the 6-chloro/7-methoxy motif present on the quinolone core appears to prevent interaction with the human host cytochrome *bc*_1_ complex as all such derivatives were devoid of inhibitory activity at concentrations as high as 10 μM, consistent with our previous findings.^6,22^

**Table 7:** Comparative Cytotoxicity Assessment for **ELQ-596** and **ELQ-300**^a^

**compound**	**cytotoxicity, nM HepG2 cells**
**ELQ-300**	>10,000
**ELQ-596**	>10,000

a Cytotoxicity experiments were performed in the medium in which glucose was substituted by galactose to reverse the Crabtree effect.

### Structure–Activity Profiling of 3-Biaryl-ELQs vs Drug-Sensitive and Multidrug-Resistant *P. falciparum*

Because of the outstanding activity that we observed with **ELQ-596**, we began to evaluate analogs in an attempt to optimize ELQs with the 3-position biaryl structural feature. In Table 1, we present results from our ongoing structure–activity profiling of 3-biaryl-ELQs for antiplasmodial activity. Notice that the 5,7-difluoro substitution pattern on the 3-biaryl-quinolone core (**ELQ-601**) is associated with weakened antiplasmodial activity relative to the corresponding diphenylether (**ELQ-400**) that exhibits IC_50_’s in drug-sensitive strains in the subnanomolar range. Given that the 5,7-difluoro substitution pattern is known to direct Q_o_ site targeting (inferred from cross resistance in the Tm90-C2B strain), this finding indicates that the rigid biphenyl side chain is better suited to ELQs that target the Q_i_ site. For most of this series, the 2-methyl-6-halo-7-methoxy-4(1*H*)-quinolone core was retained, whereas the nature and positioning of R-groups on the outermost aromatic ring of the 3-biaryl feature were varied (Table 2). Many of these structural variants exhibited low nM IC_50_ values against drug-sensitive and multidrug-resistant *P. falciparum* strains D6 and Dd2, respectively, as well as the ATV^R^ clinical isolate Tm90-C2B. With electron-withdrawing groups at the para position of the outermost ring, e.g., −OCHF_2_, CF_3_, CHF_2_, CN, Cl, and SF_5_, IC_50_ values ranged from 0.6 to 25.2 nM with a high degree of selectivity for *P. falciparum* to the reference mammalian cell line HepG2. The most active molecules in this series apart from **ELQ-596** were the para chloro analog **ELQ-637**, the para CHF_2_ variant **ELQ-659**, the para methyl analog **ELQ-603**, the para *tert*-butyl analog **ELQ-651**, the para 4-trifluoro methoxy phenoxy **ELQ-653**, and the 6-fluoro-7-methoxy quinolone variant **ELQ-650** (Table 1). It is noteworthy that **ELQ-650** retains significant activity against the **ELQ-300**^**R**^ D1 clone, which was also observed by Stickles et al. for the 6-fluoro-7-methoxy analog of **ELQ-300**, i.e., **ELQ-316.**(9) Substitution of the 3-biaryl side chain with simple alkoxy groups (OMe) failed to improve the in vitro activity over molecules with hydrophobic electron-withdrawing substituents; however, the preparation of similar molecules with electron-donating R-groups of varying strength and polarity is needed to reach a definitive conclusion.

A few variants were made with a hydrophobic electron-withdrawing moiety at the meta position of the outer ring. Placing a −CF_3_ (**ELQ-646**), −OCF_3_ (**ELQ-604**), or −SF_5_ (**ELQ-662**) at the meta position also gave impressively low to sub-nM IC_50_ values that approached or even bettered the strikingly low nM values observed for the early frontrunner in this series, **ELQ-596**. It is noteworthy that the degree of crystallinity of **ELQ-646** appears to be decreased relative to **ELQ-596** as the melting point recorded for the former (330 °C) is 35 °C lower than for the current series lead. An evaluation of the effect of ortho substituents on crystallinity, solubility, and antiplasmodial activity is currently under way.

Overall, the data from this limited series of 3-biaryl-ELQs are consistent with the idea that positioning of a hydrophobic electron-withdrawing substituent on the outermost ring of the lateral side chain and the overall lipophilicity of the drug (with an optimum of cLog*P* ∼ 5.0) are significant features influencing the intrinsic in vitro activity and in vivo efficacy of our most active cytochrome *bc*_1_ Q_i_ site targeting 3-biaryl-ELQs.

### Structural Biology and Modeling of **ELQ-596** into *P. falciparum* cyt *bc*_1_

**Figure 3:**
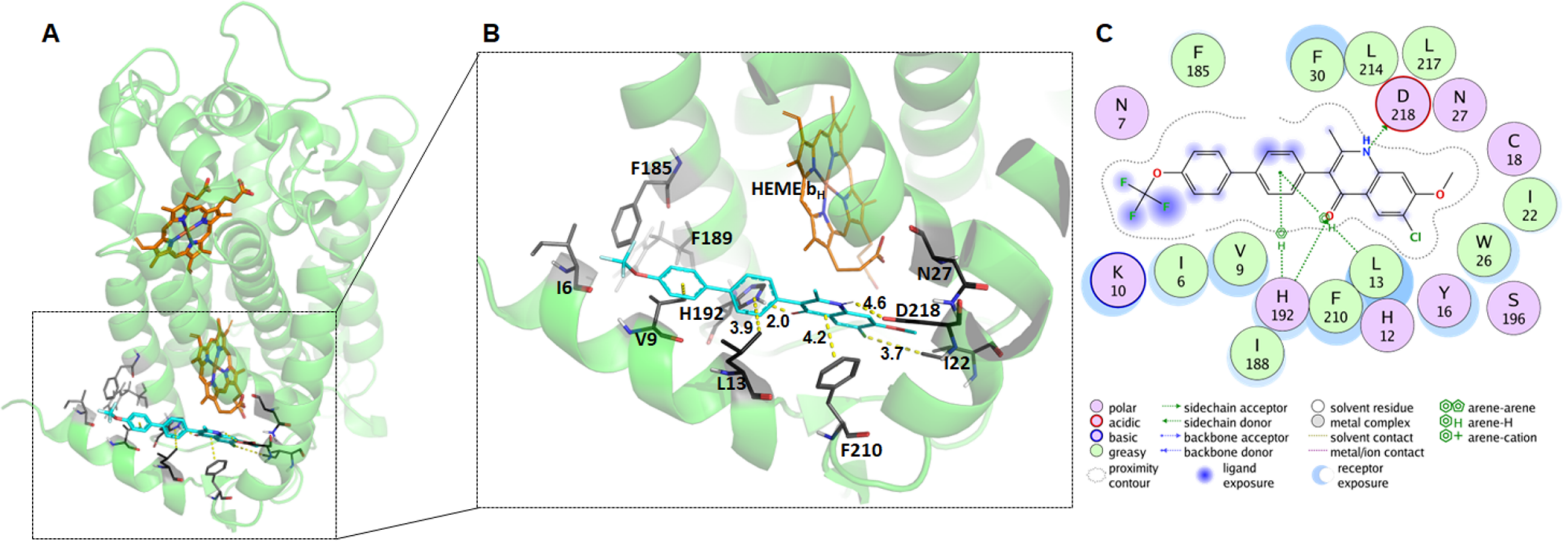
Docked complex of **ELQ-596** at Qi site of the *P. falciparum* cytochrome *b* subunit of the cyt *bc*_1_ complex. (A, B) The cytochrome *b* subunit is represented as ribbons and colored green, heme is represented in the stick model and colored orange, **ELQ-596** is colored by atom type (C: cyan, O: red, N: blue, Cl: dark green), and interacting residues are colored gray and labeled. The distance of interaction is shown in yellow dotted lines (C) Schematic mapping of **ELQ-596** interactions with the cytochrome *b* subunit is shown with scheme legend below generated using Molecular Operating Environment (MOE).

As described in the Materials and Methods section, we built a computer simulated model of the *P. falciparum* cytochrome *b* using the bovine heart cytochrome *bc*_1_ crystal structure as a template for the primary sequence of the parasite’s cytochrome *b*. Homology modeling and simulated docking of ELQs to the modeled protein were performed using the Molecular Operating Environment (MOE) software together with the docking software. The docking score for all ELQ compounds is listed in Table 2. A scatter plot of the docking score against the pIC_50_ (−log(IC_50_, M)) values shows a positive correlation for the D6 and Dd2 strains with correlation coefficients of 0.73 and 0.74, respectively (Figure S5). Analysis of the docked poses of ELQ ligands with the *P. falciparum* cyt b subunit shows that they are bound in a common pose at the Q_i_ site with the quinolone ring facing deep inside the cavity. The docking pose and the ligand interaction map of the representative ligand **ELQ-596** show that this quinolone ring is stabilized by several favorable interactions including the two H-bonds between the amine group of the quinolone ring system and Asp218 and between the carbonyl oxygen and His192. The central aromatic ring in the biaryl group is also stabilized by arene-H interactions with the side chain atoms of Leu13 and His192 residues (Figure 3).

## Concluding Remarks and Future Directions

We had synthesized biaryl-ELQs previously, and many were added to our ELQ chemical library during the MMV quinolone collaboration over a decade ago. However, none of these molecules appeared to be equal or superior to **ELQ-300**. Some of these biaryl-ELQs appeared in publications by us^23^ and by the Manetsch group^24^ after conclusion of our collaboration. Surprisingly, **ELQ-596** was not made by either team until now, and based on our findings of enhanced activity, it seemed important to reexamine modifications to the outermost aryl group of the 3-position biaryl substituent. This has been pursued in an attempt to increase inhibitor affinity for the *Pf* cyt *bc*_1_ Q_i_ site and to improve overall antimalarial performance. To date, we have used the Craig Plot^25^ to guide our placement of substituents on the outermost ring, with isosteric replacement of groups varying in hydrophobicity as well as electronic and steric properties.

Our initial goal in this work was to produce a powerful backup to **ELQ-331**. We hypothesized that the enhanced activity of **ELQ-596** compared to **ELQ-300** is due to the substituted biphenyl group making more favorable contacts within the targeted Q_i_ site of *P. falciparum* cytochrome *bc*_1_ complex. Because the cytochrome *bc*_1_ complex of *P. falciparum* had yet to be crystallized and thus a definitive structure was not available to make informed predictions, we pursued an iterative medicinal chemistry approach to focus primarily on making changes to the R-group(s) of the outermost aryl ring of the biphenyl, hoping to improve inhibitor binding affinity at the Q_i_ site. Greater inhibitor binding affinity at the Q_i_ site should produce ELQs with even more pronounced antimalarial activity.

**Figure 4:**
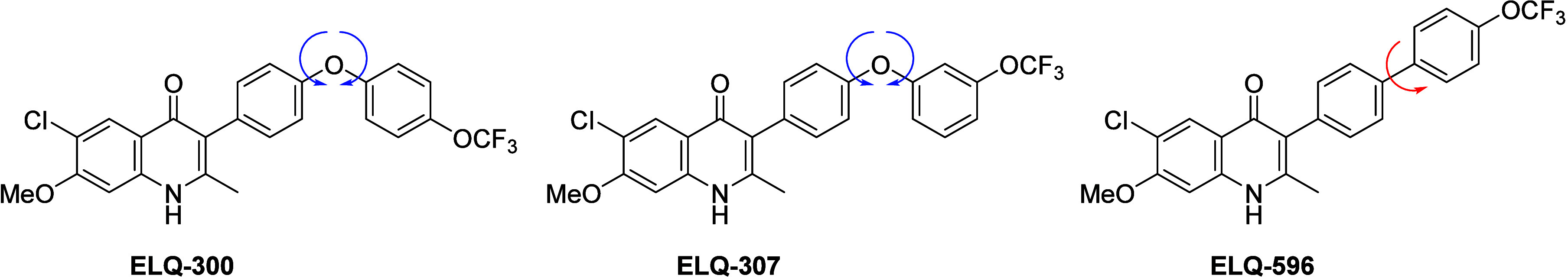
Structures of **ELQ-300**, **ELQ-307**, and **ELQ-596**. Arrows show the rotational flexibility of selected ELQs with and without the ethereal oxygen atom in the 3-position side chain.

**Figure 5:**
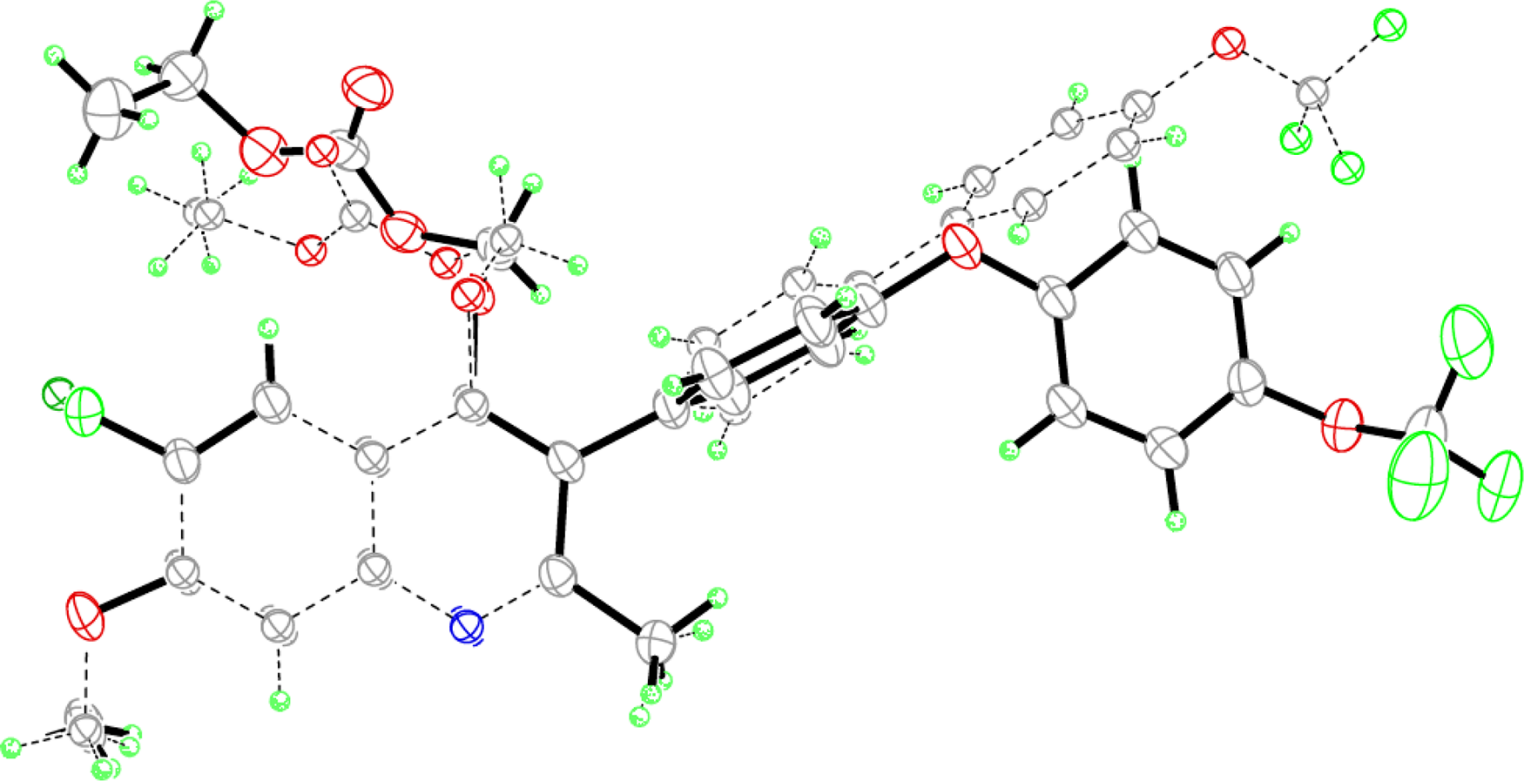
This is an overlay of the ORTEP (Oak Ridge Thermal Ellipsoid Plot) diagrams of **ELQ-331** (bold lines) and **ELQ598** (dashed lines). Ellipsoids are drawn at the 30% probability level. These structures were generated from X-ray diffraction patterns of **ELQ-331** and **ELQ-598** crystals. The image shows that the quinoline core nucleus remains a constant structural feature of both molecules whereas the rigid biaryl ring system of **ELQ-598** projects into chemical space not occupied by the angular diphenylether side chain of **ELQ-331**.

It seems relevant and important at this point to explain what compelled us to make **ELQ-596**, i.e., after we realized that it was not in our ELQ library. We previously made **ELQ-307** (Figure 4) about a month after discovering **ELQ-300** and were intrigued by its increased potency (i.e., IC_50_’s ≤ 0.03 nM vs D6, Dd2, and Tm90-C2B) relative to the new project lead candidate. Unfortunately, **ELQ-307** was metabolically unstable and inferior to **ELQ-300** in vivo against malaria in mice. But results from our in vitro study of **ELQ-307** led us to believe that improvements in intrinsic potency were possible and that the 3-position side chain of **ELQ-307** strikes at the metaphorical “bull’s eye” in the *Pf* cyt. *bc*_1_ target. We felt that we were narrowly missing that “bull’s eye” target with **ELQ-300**, which exhibited higher IC_50_ values matched with excellent metabolic stability. We prepared metabolically stable versions of **ELQ-307** with enhanced flexibility in both the diaryl ether ring system and the trifluoromethoxy substituent, but all of these derivatives (19e and 19f^22^) gave reduced activity relative to **ELQ-300** both in vitro and in vivo. Modeling of **ELQ-300** reminded us that the diaryl ether allows the outer ring to sweep around at an angle from the ethereal oxygen atom (∼109°) tracing a circle with the OCF_3_ substituent and that somewhere within that circle lies the “bull’s eye” that we want to target (Figure 5). Leaving flexibility aside and with inspiration from the GSK pyridone project,^26^ we decided to revisit 3-position biaryl side chain projections that could more directly aim at the “bull’s eye”. Future analyses will assess **ELQ-596** for inhibitor potency against the suspected *Pf* cyt *bc*_1_ target. However, the results shown here demonstrate that it displays superior antiplasmodial activity and in vivo antimalarial efficacy together with a more extended pharmacokinetics profile than were achieved by its predecessor. In a separate study that is detailed in a recent issue, **ELQ-596** shows impressive curative efficacy in two different models of murine babesiosis when delivered as the prodrug **ELQ-598**.^27^

At this juncture, we cannot predict whether **ELQ-598** will be advanced as a “backup” to **ELQ-331**, as a replacement, or as a “next-generation antimalarial ELQ” for low-dose weekly or monthly protection against malaria or for an even longer duration of protection afforded by sustained-release injectable or implantable formulations. Although it remains to be proven, the potential translational value of this discovery is that **ELQ-598** (or another more effective drug from this series) could be used as effectively as **ELQ-331** in humans but at a lower weekly dose (perhaps ∼10 mg), thereby increasing therapeutic efficiency, improving outcomes, and minimizing risk by decreasing exposure to the drug. And with a longer bloodstream half-life for **ELQ-596**, its prodrug **ELQ-598** may prove useful in providing once-monthly protection from malaria. Moreover, we believe that structural modifications to **ELQ-596** may further enhance selectivity for the Q_i_ site of the *P. falciparum* cyt *bc*_1_ complex, which may in turn improve on-target inhibitory potency and in vivo efficacy with corresponding reductions in dose, drug exposure, and cost.

## Materials and Methods

### Chemical Synthesis Procedures

Unless otherwise stated, all chemicals and reagents were from Sigma-Aldrich Chemical Co. in St. Louis, MO (USA), Combi-Blocks, San Diego (CA), or TCI America, Portland (OR), and were used as received. 4(1*H*)-Quinolone **1** and 4,4,5,5-tetramethyl-2-(4-(4-(trifluoromethoxy)phenoxy)phenyl)-1,3,2-dioxaborolane (**15k**) were obtained as previously reported.^12^ Melting points were obtained in the Optimelt Automated Melting point system from Stanford Research Systems, Sunnyvale, CA (USA). Analytical TLC utilized DC Kieselgel 60F_254_ precoated silica gel plates, and spots were visualized under 254 nm UV light. GC–MS was obtained using an Agilent Technologies 7890B gas chromatograph (30 m, DBS column set at either 100 or 200 °C for 2 min, then at 30 °C/min to 300 °C with inlet temperature set at 250 °C) with an Agilent Technologies 5977A mass-selective detector operating at 70 eV. Flash chromatography over silica gel column was performed using an Isolera One flash chromatography system from Biotage, Uppsala, Sweden. Unless otherwise noted, the default TLC method as implemented by the machine was used in the gradient of the eluting solvent. ^1^H NMR spectra were obtained using a Bruker 400 MHz Avance NEO NanoBay NMR spectrometer operating at 400.14 MHz. The NMR raw data were analyzed using the iNMR Spectrum Analyst software. ^1^H chemical shifts are reported in parts per million (ppm) relative to internal tetramethylsilane (TMS) standard or residual solvent peak. Coupling constant values (*J*) are reported in hertz (Hz). Decoupled ^19^F operating at 376 MHz was also obtained for compounds containing fluorine (data not shown). HPLC analyses were performed using an Agilent 1260 Infinity instrument with detection at 254 nm and a Phenomenex Luna 5 μm C8(2) 100 Å reverse phase LC column 150 × 4.6 mm at 40 °C and eluted with a gradient of A/B at 25:75% to A/B at 10:90% (A: 0.05% formic acid in Milli-Q water, B: 0.05% formic acid in methanol). High-resolution mass spectrometry (HRMS) was performed using a high-resolution (30,000) Thermo LTQ-Orbitrap Discovery hybrid mass spectrometry instrument (San Jose, CA) equipped with an electrospray ionization source operating in the positive or negative ion mode. The Orbitrap was externally calibrated prior to data acquisition, allowing accurate mass measurements for [M + H]^+^ ions to be obtained within 4 ppm. All compounds were at least >95% pure for in vitro testing and >98% pure for in vivo testing as determined by GC–MS, ^1^H NMR, and HPLC.

#### 4,6-Dichloro-3-iodo-7-methoxy-2-methylquinoline (**2**)

A stirred solution of 4(1*H*)-quinolone **1** (10.0 g, 28.6 mmol, 1 equiv) and POCl_3_ (14 mL, 146 mmol, 5.1 equiv) in DCM (100 mL) was refluxed for 72 h. After cooling to room temperature, the mixture was filtered, and the precipitate was washed with DCM (3 × 5 mL) and air-dried to give pure **2** (9.8 g, 93% yield) as a white powder. GC–MS shows one peak M^+^ = 366.9 (100%). ^1^H NMR (400 MHz; DMSO-*d*_6_): δ 8.20 (s, 1H), 7.59 (s, H), 4.04 (s, 3H), 2.92 (s, 3H).

#### 4,4,5,5-Tetramethyl-2-(4′-(trifluoromethoxy)-[1,1′-biphenyl]-4-yl)-1,3,2-dioxaborolane (**4**)

A stirred mixture of 4-bromo-4(trifluoromethoxy)biphenyl **3** (2.51 g, 7.91 mmol, 1.0 equiv), bis(pinacolato)diboron (4.43 g, 17.4 mmol, 2.2 equiv), KOAc (3.89 g, 39.6 mmol, 5.0 equiv), and Pd(dppf)Cl_2_ (583 mg, 0.78 mmol, 0.10 equiv) in DMF (30 mL) was deoxygenated by bubbling argon through the reaction mixture for 15 min. The stirred reaction mixture was then heated at 80 °C under argon for 48 h until no more **3** remained as determined by GC–MS. The reaction was cooled to room temperature, and DMF was removed in vacuo. The resulting black oil was resuspended in EtOAc (100 mL), filtered over Celite, and washed with additional EtOAc (3 × 100 mL). The resulting filtrate was transferred to a separatory funnel, washed with H_2_O (3 × 150 mL) and saturated NaCl solution (150 mL), dried with MgSO_4_, and concentrated to dryness. The mixture was purified by flash chromatography over silica gel using a gradient of ethyl acetate/hexane as the eluting solvent mixture to give **4** (2.044 g, 71% yield) as a white solid. GC–MS shows one peak M^+^ = 364.1 (40%); 264.0 (100%). ^1^H NMR (400 MHz; CDCl_3_): δ 7.90–7.88 (m, 2H), 7.64–7.60 (m, 2H), 7.58–7.56 (m, 2H), 7.29 (dd, J = 8.8, 0.9 Hz, 2H), 1.37 (s, 12H).

#### 4,6-Dichloro-7-methoxy-2-methyl-3-(4′-(trifluoromethoxy)-[1,1′-biphenyl]-4-yl)quinoline (**5**)

A stirred mixture of **4** (255 mg, 0.70 mmol, 1.0 equiv), **2** (262 mg, 0.71 mmol, 1.0 equiv), 2 M K_2_CO_3_ (0.70 mL, 1.40 mmol, 2.0 equiv), and Pd(dppf)Cl_2_ (29 mg, 0.04 mmol, 0.05 equiv) in DMF (5 mL) was deoxygenated by bubbling argon through the reaction mixture for 15 min. The stirred reaction mixture was then heated at 80 °C under argon for 24 h until no more starting material **4** remained as determined by TLC. The reaction was cooled to room temperature, and DMF was removed in vacuo. The resulting black solid was resuspended in EtOAc (50 mL) and filtered over Celite. The resulting filtrate was transferred to a separatory funnel, washed with H_2_O (3 × 50 mL) and saturated sodium chloride solution (50 mL), dried with MgSO_4_, and concentrated to dryness. The mixture was purified by flash chromatography over silica gel using a gradient of ethyl acetate/hexane as the eluting solvent mixture to give **5** (0.238 g, 71% yield) as a white solid. GC–MS shows one peak M^+^ = 477.1 (100%). ^1^H NMR (400 MHz; DMSO-*d*_6_): δ 8.19 (s, 1H), 7.93–7.86 (m, 4H), 7.65 (s, 1H), 7.50 (d, J = 8.5 Hz, 4H), 4.07 (s, 3H), 2.43 (s, 3H).

#### 6-Chloro-7-methoxy-2-methyl-3-(4′-(trifluoromethoxy)-[1,1′-biphenyl]-4-yl)quinolin-4(1*H*)-one (**ELQ-596**)

Following the general procedure for the hydrolysis of the 4-chloro quinolines, a mixture of 4,6-dichloro-7-methoxy-2-methyl-3-(4′-(trifluoromethoxy)-[1,1′-biphenyl]-4-yl)quinoline (193 mg, 0.40 mmol, 1 equiv), KOAc (409 mg, 4.17 mmol, 10 equiv), and glacial acetic acid (3 mL) was heated for 16 h. After cooling to room temperature, the reaction mixture was poured into ice water (20 mL). The resulting precipitate was filtered; washed with water (3 × 10 mL), acetone (2 × 10 mL), DCM (2 × 10 mL), and hexane (10 mL); and air-dried to give pure **ELQ-596** (184 mg, 85% yield) as a white solid. ^1^H NMR (400 MHz; DMSO-*d*_6_): δ 11.69 (s, 1H), 8.01 (s, 1H), 7.87–7.83 (m, 2H), 7.72–7.69 (m, 2H), 7.50–7.44 (m, 2H), 7.38–7.35 (m, 2H), 7.09 (s, 1H), 3.97 (s, 3H), 2.26 (s, 3H); mp 348.1–351.9 °C (decomposed).

#### Ethyl 2-(4′-(Trifluoromethoxy)-[1,1′-biphenyl]-4-yl)acetate (**7**)

A stirred mixture of ethyl 2-(4-bromophenyl)-acetate **5** (48.6 g, 200.0 mmol, 1.0 equiv), (4-(trifluoromethoxy)phenyl)boronic acid (49.5 g, 240.0 mmol, 1.2 equiv), K_2_CO_3_ (55.2 g, 400.0 mmol, 2 equiv), and (Pd(dppf)Cl_2_) (7.30 g, 10.0 mmol, 0.05 equiv) in DMF (500 mL) was deoxygenated by bubbling argon through the reaction mixture for 15 min. The stirred reaction mixture was then heated at 80 °C under argon for 18 h until no more starting material **6** remained as determined by GC–MS. The reaction was cooled to room temperature and filtered through Celite, and DMF was removed in vacuo. The resulting black oily solid was resuspended in DCM (500 mL) and stirred vigorously at room temperature for 30 min, filtered through Celite, concentrated to dryness, and purified by flash chromatography over silica gel using a gradient of ethyl acetate/hexane as the eluting solvent mixture to give **7** (50.1 g, 77% yield) as a white solid. GC–MS shows one peak M^+^ = 324.1 (42%); 251.2 (100%). ^1^H NMR (400 MHz; CDCl_3_): δ 7.63–7.59 (m, 2H), 7.56–7.53 (m, 2H), 7.41–7.38 (m, 2H), 7.32–7.29 (m, 2H), 4.21 (q, *J* = 7.1 Hz, 2H), 3.69 (s, 2H), 1.32–1.29 (t, *J* = 7.1 Hz, 3H).

#### Ethyl 3-Acetoxy-2-(4-bromophenyl)but-2-enoate (**8a**)

Temperatures given were recorded by an internal thermometer. A stirred solution of dry THF (250 mL) and HMDS (179.5 g, 231 mL, 1.11 mol, 2.7 equiv) under argon was cooled to −20 °C in an 75% ethylene glycol, 25% ethanol, and dry ice bath. While monitoring the temperature to ensure that it did not exceed −10 °C, *n*-butyl-lithium (2.5 M) in hexane (*n*-BuLi) (445 mL, 1.11 mol, 2.7 equiv) was added dropwise. The temperature of the solution was then lowered to −20 °C and allowed to stir for 15 min. Then a solution of **6** (100.0 g, 412.0 mmol, 1.0 equiv) in THF (250 mL) was slowly added dropwise while maintaining the temperature at or below −10 °C, and the solution was stirred for 30 min at −10 °C. While maintaining the temperature at or below −10 °C, acetic anhydride (126.2 g, 142 mL, 1.236 mol, 3.0 equiv) was added dropwise. Then the solution was allowed to slowly warm up to room temperature and heated at 26 °C for 72 h. A gel was formed and was poured into saturated ammonium chloride solution (500 mL). The organic layer was separated from the aqueous layer and extracted with ethyl acetate (2 × 500 mL). The organic layers were combined, washed with water (250 mL) and saturated NaCl solution (250 mL), dried with MgSO_4_, and concentrated to give 138.5 g (>100% yield) of a brown oil. GC–MS analysis showed one major peak (100%) with M^+^ = 326 (2%), 238 (100%); one minor peak (27%) with M^+^ = 326 (2%), 238 (100%); and another minor peak (8%, corresponding to **6**) with M^+^ = 242 (25%), 168.9 (100%). The peaks with M^+^ = 326 correspond to the stereoisomers E and Z of the desired product **8a**. Two-dimensional NMR analysis using NOESY did not provide unambiguous assignment of the two stereoisomers. The percent of the major stereoisomer relative to the minor stereoisomer was estimated to be 80% by GC–MS. The product was used without further purification in the next step.

For analysis and characterization purposes, the two stereoisomers were purified by flash chromatography using hexane and ethyl acetate (5 to 15% gradient).

NMR of the major stereoisomer of **8a**: ^1^H NMR (400 MHz; CDCl_3_): δ 7.51–7.48 (m, 2H), 7.18–7.15 (m, 2H), 4.14 (q, *J* = 7.1 Hz, 2H), 2.23 (s, 3H), 1.88 (s, 3H), 1.19 (t, *J* = 7.1 Hz, 3H).

NMR of the minor stereoisomer of **8a**: ^1^H NMR (400 MHz; CDCl_3_): δ 7.47–7.44 (m, 2H), 7.08–7.05 (m, 2H), 4.20 (q, *J* = 7.1 Hz, 2H), 2.40 (s, 3H), 1.88 (s, 3H), 1.23 (t, *J* = 7.1 Hz, 3H).

#### Ethyl 3-Acetoxy-2-(4′-(trifluoromethoxy)-[1,1′-biphenyl]-4-yl)but-2-enoate (**8b**)

Temperatures given were recorded by an internal thermometer. A stirred solution of dry THF (200 mL) and HMDS (67.3 g, 417.0 mmol, 2.7 equiv) under argon was cooled to −20 °C in 75% ethylene glycol, 25% ethanol, and dry ice bath. While monitoring the temperature to ensure that it did not exceed −10 °C, *n*-butyl-lithium (2.5 M) in hexane (*n*-BuLi) (166.7 mL, 417.0 mmol, 2.7 equiv) was added dropwise, and after 10 min at −20 °C, a solution of **7** (50.0 g, 154.0 mmol, 1.0 equiv) in THF (150 mL) was added dropwise. After stirring for 35 min at −15 to −10 °C, acetic anhydride (47.3 g, 53.1 mL, 463.0 mmol, 3.0 equiv) was added dropwise while monitoring the temperature to ensure that it did not exceed −10 °C. The solution was then allowed to gradually warm to room temperature, when it turned into a light-yellow gel. After stirring for 72 h at 26 °C, the mixture was poured into saturated ammonium chloride solution (300 mL). The organic layer was separated from the aqueous layer and extracted with ethyl acetate (2 × 200 mL). The organic layers were combined, washed with saturated NaCl solution (150 mL), dried with MgSO_4_, and concentrated to give 67.4 g (>100% yield) of a brown oil. GC–MS analysis showed one major peak (100%) with M^+^ = 408 (3%), 320 (100%); one minor peak (5%) with M^+^ = 408 (3%), 320 (100%); and another minor peak (5% corresponding to the starting material **7**) with M^+^ = 324 (42%), 251 (100%). The peaks with M^+^ = 408 correspond to the mixture of the stereoisomers E and Z of the desired product **8b**. Two-dimensional NMR analysis using NOESY did not provide unambiguous assignment of the two stereoisomers. The percent of the major stereoisomer relative to the minor stereoisomer was estimated to be 95% by GC–MS. The product can be used without further purification in the next step.

For analysis and characterization purposes, the two stereoisomers were purified by flash chromatography using hexane and ethyl acetate (5 to 50% gradient).

NMR of the major stereoisomer of **8b**: ^1^H NMR (400 MHz; CDCl_3_): δ 7.66–7.62 (m, 2H), 7.60–7.57 (m, 2H), 7.41–7.38 (m, 2H), 7.32–7.30 (m, 2H), 4.20 (q, *J* = 7.1 Hz, 2H), 2.27 (s, 3H), 1.98 (s, 3H), 1.25 (t, *J* = 7.1 Hz, 3H).

NMR of the minor stereoisomer of **8b**: ^1^H NMR (400 MHz; CDCl_3_): δ 7.66–7.62 (m, 2H), 7.57–7.54 (m, 2H), 7.32–7.29 (m, 4H), 4.25 (q, *J* = 7.1 Hz, 2H), 2.44 (s, 3H), 1.90 (s, 3H), 1.27 (t, *J* = 7.1 Hz, 3H).

#### Ethyl 2-(4-Bromophenyl)-3-oxobutanoate (**9a**)

A stirred solution of the bis-acylated **8a** (138.5 g, 424.0 mmol, 1 equiv) in glacial acetic acid (280 mL) and *p*-TsOH monohydrate 98% (8.06 g, 42.4 mmol, 0.1 equiv) was heated at 100 °C. After 3 h, no more starting material **8a** was detected by GC–MS. The dark brown solution was cooled to room temperature and concentrated in vacuo. After most of the acetic acid was eliminated, hexane (2 × 100 mL) was added to the brown oil and concentrated again to give 131.3 g of **9a** as a dark brown oil. Because this material still contained 8.06 g of *p*-TsOH, the yield of **9a** was 123.2 g (>100% yield). The product can be used without purification in the following Conrad–Limpach reaction. Both the keto and enol forms can be detected by ^1^H NMR.

#### Ethyl 3-Hydroxy-2-(4′-(trifluoromethoxy)-[1,1′-biphenyl]-4-yl)but-2-enoate (**9b**)

A stirred solution of the bis-acylated **8b** (67.4 g, 165.0 mmol, 1 equiv) in glacial acetic acid (140 mL) and *p*-TsOH monohydrate 98% (3.14 g, 16.5 mmol, 0.1 equiv) was heated at 100 °C. After 3 h, no more starting material **8b** was detected by GC–MS. Of note, the β-keto ester **9b** decomposed in the injection port of the mass spectrometer to give a major peak with M^+^ = 294 (32%), 251 (100%). The dark brown solution was cooled to room temperature and concentrated under a vacuum. After most of the acetic acid was eliminated, hexane (2 × 100 mL) was added to the brown oil and concentrated again to give 61.8 g of **9b** as a dark brown oil. Because this material still contained 3.14g of p-TsOH, the yield of **9b** was 58.7 g (97% yield). The product can be used without purification in the following Conrad–Limpach reaction. Both the keto and enol forms can be detected by ^1^H NMR.

#### General Procedure for the Preparation of Schiff Bases (**11a**–**d** and **12**)

For use in these reactions, a benzene stock solution was prepared at a concentration of 2.5 mM of **9a** or **9b** (per 10 mL solvent, 0.92 g of **9a** or **9b** that already contained 0.1 equiv of *p*-TsOH, 0.25 mmol, from the previous reaction). A stirred solution of a substituted aniline (**10a**–**d**) in benzene and an aliquot of the stock solution of β-keto ester **9a** or **9b** was heated at reflux for 24–72 h using a Dean–Stark trap to continuously remove water azeotropically and monitored for the disappearance of β-keto ester **9a** or **9b** by GC–MS. The solution was then concentrated in vacuo to give the product Schiff bases (**11a**–**d** and **12**) as yellow-brown, highly viscous oils.

#### General Procedure for the Conrad–Limpach Reaction (ELQ)

The intermediate Schiff base (**11a**–**d** and **12**) was diluted with 5 mL of warm Dowtherm A and added to 65 mL of boiling Dowtherm A (250 °C) in portions over approximately 5 min with vigorous stirring to maintain the boiling of Dowtherm A. The mixture was kept at boiling for another 5 min, allowed to cool to room temperature, and diluted with hexane (250 mL), resulting in the formation of a precipitate that was filtered and washed with ethyl acetate and acetone until a colorless filtrate was obtained.

#### 2-Methyl-3-(4′-(trifluoromethoxy)-[1,1′-biphenyl]-4-yl)quinolin-4(1*H*)-one (**ELQ-649**)

Following the general procedure for the preparation of the Schiff base, a mixture of aniline **10a** (0.47 g, 5.0 mol, 1 equiv), benzene (125 mL), and β-keto ester **9b** (5.0 mmol, 1 equiv) containing *p*-TsOH (0.5 mmol, 0.1 equiv) was heated at reflux for 24 h. The general procedure for the Conrad–Limpach reaction was followed to give **ELQ-649** (0.64 g, 32% yield) after crystallization using DMF as a white powder. ^1^H NMR (400 MHz; DMSO-*d*_6_): δ 11.68 (s, 1H), 8.10 (dd, *J* = 8.1, 1.5 Hz, 1H), 7.87–7.83 (m, 2H), 7.73–7.70 (m, 2H), 7.65 (ddd, *J* = 8.4, 6.9, 1.5 Hz, 1H), 7.56–7.54 (m, 1H), 7.49–7.46 (m, 2H), 7.39–7.36 (m, 2H), 7.30 (ddd, *J* = 8.1, 6.9, 1.1 Hz, 1H), 2.29 (s, 3H); mp 362.6–365.5 °C (decomposed). HRMS calculated for C_23_H_17_F_3_N_1_O_2_ [M + H]^+^ = 396.1206, observed for [M + H]^+^ = 396.1194.

#### 6-Chloro-7-methoxy-2-methyl-3-(4′-(trifluoromethoxy)-[1,1′-biphenyl]-4-yl)quinolin-4(1*H*)-one (**ELQ-596**)

Following the general procedure for the preparation of the Schiff base, a mixture of aniline **10b** (0.78 g, 5.0 mmol, 1 equiv), benzene (125 mL), and β-keto ester **9b** (5.0 mmol, 1 equiv) containing *p*-TsOH (0.5 mmol, 0.1 equiv) was heated at reflux for 72 h. The general procedure for the Conrad–Limpach reaction was followed to give **ELQ-596** (0.77 g, 34% yield) as a white powder. ^1^H NMR (400 MHz; DMSO-*d*_6_): δ 11.69 (s, 1H), 8.01 (s, 1H), 7.87–7.83 (m, 2H), 7.72–7.69 (m, 2H), 7.50–7.44 (m, 2H), 7.38–7.35 (m, 2H), 7.09 (s, 1H), 3.97 (s, 3H), 2.26 (s, 3H); mp 348.1–351.9 °C (decomposed). HRMS calculated for C_24_H_18_Cl_1_F_3_N_1_O_3_ [M + H]^+^ = 460.0922, observed for [M + H]^+^ = 460.0915.

#### 6-Fluoro-7-methoxy-2-methyl-3-(4′-(trifluoromethoxy)-[1,1′-biphenyl]-4-yl)quinolin-4(1*H*)-one (**ELQ-650**)

Following the general procedure for the preparation of the Schiff base, a mixture of aniline **10c** (0.71 g, 5.0 mmol, 1 equiv), benzene (125 mL), and β-keto ester **9b** (5.0 mmol, 1 equiv) containing *p*-TsOH (0.5 mmol, 0.1 equiv) was heated at reflux for 46 h. The general procedure for the Conrad–Limpach reaction was followed to give **ELQ-650** (0.61 g, 28% yield) as a white powder. ^1^H NMR (400 MHz; DMSO-*d*_6_): δ 11.66 (s, 1H), 7.86–7.83 (m, 2H), 7.73–7.69 (m, 3H), 7.50–7.44 (m, 2H), 7.37–7.35 (m, 2H), 7.11 (d, *J* = 7.4 Hz, 1H), 3.96 (s, 3H), 2.26 (s, 3H); mp 369.4–370.6 °C (decomposed). HRMS calculated for C_24_H_18_F_4_N_1_O_3_ [M + H]^+^ = 444.1217, observed for [M + H]^+^ = 444.1208.

#### 5,7-Difluoro-2-methyl-3-(4′-(trifluoromethoxy)-[1,1′-biphenyl]-4-yl)quinolin-4(1*H*)-one (**ELQ-601**)

Following the general procedure for the preparation of the Schiff base, a mixture of aniline **10d** (0.71 g, 5.0 mmol, 1 equiv), benzene (125 mL), and β-keto ester **9b** (5.0 mmol, 1 equiv) containing *p*-TsOH (0.5 mmol, 0.1 equiv) was heated at reflux for 72 h. The general procedure for the Conrad–Limpach reaction was followed to give **ELQ-601** (0.80 g, 37% yield) as a white powder. ^1^H NMR (400 MHz; DMSO-*d*_6_): δ 11.76 (s, 1H), 7.87–7.83 (m, 2H), 7.73–7.69 (m, 2H), 7.48–7.46 (m, 2H), 7.36–7.32 (m, 2H), 7.11–7.08 (m, 1H), 7.04 (ddd, *J* = 11.9, 9.6, 2.4 Hz, 1H), 2.22 (s, 3H); mp 359.9–362.3 °C (decomposed). HRMS calculated for C_23_H_15_F_5_N_1_O_2_ [M + H]^+^ = 432.1017, observed for [M + H]^+^ = 432.1011.

#### 3-(4-Bromophenyl)-6-chloro-7-methoxy-2-methylquinolin-4(1*H*)-one (**13**)

For this reaction, the general procedure for the preparation of the Schiff base was followed except that the stock solution of β-keto ester in benzene was not used. A mixture of aniline **10b** (68.0 g, 432 mmol, 1 equiv), cyclohexane (400 mL), and β-keto ester **9a** (123.2 g, 432 mmol, 1 equiv) in admixture with *p*-TsOH (8.06 g, 42.4 mmol, ∼0.1 equiv) was heated at reflux for 21 h to produce Schiff base **12**. The reaction solution was filtered to remove a small amount of black insoluble solid, and after removal of the solvent under vacuo, the crude Schiff base was used without further purification. The general procedure for the Conrad–Limpach reaction was then followed using Dowtherm A (80 mL) to dilute Schiff base **12** with warming followed by addition to boiling Dowtherm A (400 mL). (The solution of **12** in Dowtherm A had to be kept warm to prevent crystallization.) Upon cooling while stirring, a precipitate was formed. When at room temperature, the solid was suction filtered and washed with ethyl acetate (3 × 50 mL) and then acetone (3 × 50 mL) and air-dried to give pure **13** (104.0 g, 63.5% yield) as a beige solid. ^1^H NMR (400 MHz; DMSO-*d*_6_): δ 11.70 (s, 1H), 7.99 (s, 1H), 7.58–7.56 (m, 2H), 7.24–7.18 (m, 2H), 7.07 (s, 1H), 3.96 (s, 3H), 2.21 (s, 3H).

#### 3-(4-Bromophenyl)-4,6-dichloro-7-methoxy-2-methylquinoline (**14**)

4(1*H*)-Quinolone **13** (14.7 g, 39.0 mmol) was refluxed with POCl_3_ (70 mL) for 45 min. After cooling to room temperature, the solution was poured slowly over 10 min in vigorously stirred water (800 mL) and stirred for an additional 5 min. The precipitate formed was washed with water (50 mL) and acetone (2 × 25 mL) and air-dried to give pure **14** (15.7 g, 100% yield). GC–MS shows one peak M^+^ = 395 (63%), 397 (100%), 399 (47%), 401 (10%). ^1^H NMR (400 MHz; DMSO-*d*_6_): δ 8.18 (s, 1H), 7.76–7.73 (m, 2H), 7.64 (s, 1H), 7.37–7.34 (m, 2H), 4.05 (s, 3H), 2.38 (s, 3H).

#### General Procedure for the Preparation of the Biphenyl Quinolines (**16a**–**k**)

A stirred mixture of quinoline **14**; substituted phenyl boronic acids **15a**–**e**, **15g**, **15i**, **15j**, and **15l**–**m** or pinacol esters **15f**, **15h**, and **15k**; K_2_CO_3_; and Pd(dppf)Cl_2_ in DMF was deoxygenated by bubbling argon through the solution for 15 min. The stirred reaction mixture was then heated at 80 °C under argon until almost no more starting material **14** remained as determined by GC–MS. The reaction was cooled to room temperature and filtered through Celite, and DMF was removed in vacuo. The resulting black oily solid was resuspended in DCM and stirred vigorously at room temperature for 30 min, filtered through Celite, and concentrated to dryness. The residue was taken up with 3–5 mL of DCM; if all the solid was dissolved, then the product was purified by flash chromatography. In instances where the products were not completely soluble in methylene chloride, the solids were recovered by filtration and washing with DCM. The solid obtained was recrystallized to obtain the desired product, whereas the filtrates were further purified by flash chromatography to give additional material.

#### General Procedure for the Hydrolysis of the 4-Chloro Quinolines

A stirred mixture of the 4-chloro quinolines, potassium acetate (KOAc), and glacial acetic acid was heated at 120 °C in a loosely capped reaction vial for 16–24 h. After cooling to room temperature, the reaction mixture was poured into ice water. The resulting precipitate was filtered; washed with water followed by acetone, DCM, and/or hexane; and air-dried to give the pure product.

#### 4,6-Dichloro-3-(4′-chloro-[1,1′-biphenyl]-4-yl)-7-methoxy-2-methylquinoline (**16a**)

Following the general procedure for the preparation of biphenyl quinolines, a mixture of **14** (740 mg, 1.86 mmol, 1 equiv), **15a** (435 mg, 2.79 mmol, 1.5 equiv), K_2_CO_3_ (513 mg, 3.72 mmol, 2 equiv), Pd(dppf)Cl_2_ (68 mg, 0.093 mmol, 0.05 equiv), and DMF (75 mL) was heated for 16 h to give crude **16a** (911 mg) as a black solid. DCM (5 mL) was added, and the precipitate was filtered and washed with methylene chloride (2 × 5 mL) to give pure **16a** (158 mg) as a white solid. The filtrate was further purified by flash chromatography using a gradient of ethyl acetate/hexane as the eluting solvent to yield additional **16a** (99 mg) for a combined yield of **16a** (257 mg, 32% yield). GC–MS shows one peak M^+^ = 427 (100%). ^1^H NMR (400 MHz; CDCl_3_): δ 8.27 (s, 1H), 7.74–7.71 (m, 2H), 7.65–7.62 (m, 2H), 7.49–7.46 (m, 3H), 7.39–7.36 (m, 2H), 4.10 (s, 3H), 2.52 (s, 3H).

#### 4,6-Dichloro-7-methoxy-2-methyl-3-(4′-methyl-[1,1′-biphenyl]-4-yl)quinoline (**16b**)

Following the general procedure for the preparation of biphenyl quinolines, a mixture of **14** (794 mg, 2.0 mmol, 1 equiv), **15b** (326 mg, 2.4 mmol, 1.2 equiv), K_2_CO_3_ (552 mg, 4.0 mmol, 2 equiv), Pd(dppf)Cl_2_ (73 mg, 0.10 mmol, 0.05 equiv), and DMF (75 mL) was heated for 16 h to give crude **16b** (898 mg) as a black solid. The product was soluble in DCM and was purified by flash chromatography using a gradient of ethyl acetate/hexane as the eluting solvent to give pure **16b** (328 mg, 40% yield) as a white solid. GC–MS shows one peak M^+^ = 407 (100%). ^1^H NMR (400 MHz; CDCl_3_): δ 8.27 (s, 1H), 7.76–7.73 (m, 2H), 7.62–7.59 (m, 2H), 7.49 (s, 1H), 7.37–7.30 (m, 4H), 4.09 (s, 3H), 2.53 (s, 3H), 2.45 (s, 3H).

#### 3-(4′-(*tert*-Butyl)-[1,1′-biphenyl]-4-yl)-4,6-dichloro-7-methoxy-2-methylquinoline (**16c**)

Following the general procedure for the preparation of biphenyl quinolines, a mixture of **14** (794 mg, 2.0 mmol, 1 equiv), **15c** (427 mg, 2.4 mmol, 1.2 equiv), K_2_CO_3_ (552 mg, 4.0 mmol, 2 equiv), Pd(dppf)Cl_2_ (73 mg, 0.10 mmol, 0.05 equiv), and DMF (75 mL) was heated for 16 h to give crude **16c** (1.032 g) as a black solid. The product was soluble in DCM and was purified by flash chromatography using a gradient of ethyl acetate/hexane as the eluting solvent to give pure **16c** (331 mg, 37% yield) as a white solid. GC–MS shows one peak M^+^ = 450 (63%), 434 (100%). ^1^H NMR (400 MHz; CDCl_3_): δ 8.28 (s, 1H), 7.77–7.74 (m, 2H), 7.66–7.64 (m, 2H), 7.55–7.52 (m, 2H), 7.50 (s, 1H), 7.37–7.33 (m, 2H), 4.10 (s, 3H), 2.53 (s, 3H), 1.41 (s, 9H).

#### 4,6-Dichloro-7-methoxy-2-methyl-3-(4′-(trifluoromethyl)-[1,1′-biphenyl]-4-yl)quinoline (**16d**)

Following the general procedure for the preparation of biphenyl quinolines, a mixture of **14** (794 mg, 2.0 mmol, 1 equiv), **15d** (456 mg, 2.4 mmol, 1.2 equiv), K_2_CO_3_ (552 mg, 4.0 mmol, 2 equiv), Pd(dppf)Cl_2_ (73 mg, 0.10 mmol, 0.05 equiv), and DMF (75 mL) was heated for 36 h to give crude **16d** (1.12 g) as a reddish black solid. DCM (5 mL) was added, and the precipitate was filtered and washed with methylene chloride (2 × 5 mL) to give pure **16d** (320 mg, 35% yield) as a white solid. GC–MS shows one peak M^+^ = 461 (100%). ^1^H NMR (400 MHz; CDCl_3_): δ 8.28 (s, 1H), 7.83–7.80 (m, 2H), 7.78–7.76 (m, 4H), 7.50 (s, 1H), 7.42–7.40 (m, 2H), 4.10 (s, 3H), 2.53 (s, 3H).

#### 4′-(4,6-Dichloro-7-methoxy-2-methylquinolin-3-yl)-[1,1′-biphenyl]-4-carbonitrile (**16e**)

Following the general procedure for the preparation of biphenyl quinolines, a mixture of **14** (794 mg, 2.0 mmol, 1 equiv), **15e** (353 mg, 2.4 mmol, 1.2 equiv), K_2_CO_3_ (552 mg, 4.0 mmol, 2 equiv), Pd(dppf)Cl_2_ (73 mg, 0.10 mmol, 0.05 equiv), and DMF (75 mL) was heated for 36 h to give crude **16e** (769 mg) as a black solid. DCM (5 mL) was added, and the precipitate was filtered and washed with DCM (2 × 5 mL) to give **16e** (384 mg) as a white solid. The product was further recrystallized from DMF to give pure **16e** (290 mg) as a white solid. The filtrate was further purified by flash chromatography using a gradient of ethyl acetate/hexane as the eluting solvent to yield an additional **16e** (90 mg) for a combined yield of **16e** (380 mg, 45% yield). GC–MS shows one peak M^+^ = 418 (100%). ^1^H NMR (400 MHz; CDCl_3_): δ 8.26 (s, 1H), 7.82–7.79 (m, 4H), 7.77–7.75 (m, 2H), 7.49 (s, 1H), 7.44–7.41 (m, 2H), 4.09 (s, 3H), 2.51 (s, 3H).

#### 4,6-Dichloro-3-(4′-(difluoromethyl)-[1,1′-biphenyl]-4-yl)-7-methoxy-2-methylquinoline (**16f**)

Following the general procedure for the preparation of biphenyl quinolines, a mixture of **14** (794 mg, 2.0 mmol, 1 equiv), **15f** (610 mg, 2.4 mmol, 1.2 equiv), K_2_CO_3_ (552 mg, 4.0 mmol, 2 equiv), Pd(dppf)Cl_2_ (73 mg, 0.10 mmol, 0.05 equiv), and DMF (75 mL) was heated for 36 h to give crude **16f** (950 mg) as a black solid. DCM (5 mL) was added, and the precipitate was filtered and washed with DCM (2 × 5 mL) to give **16f** (500 mg) as a white solid. The product was further recrystallized from DMF to give pure **16f** (350 mg, 39% yield) as a white solid. GC–MS showed one peak M^+^ = 444 (100%). ^1^H NMR (400 MHz; CDCl_3_): δ 8.26 (s, 1H), 7.79–7.73 (m, 4H), 7.66–7.61 (m, 2H), 7.48 (s, 1H), 7.40–7.35 (m, 2H), 6.73 (t, 1H, *J* = 57 Hz), 4.08 (s, 3H), 2.51 (s, 3H).

#### 4,6-Dichloro-7-methoxy-3-(4′-methoxy-[1,1′-biphenyl]-4-yl)-2-methylquinoline (**16g**)

Following the general procedure for the preparation of biphenyl quinolines, a mixture of **14** (794 mg, 2.0 mmol, 1 equiv), **15g** (365 mg, 2.4 mmol, 1.2 equiv), K_2_CO_3_ (552 mg, 4.0 mmol, 2 equiv), Pd(dppf)Cl_2_ (73 mg, 0.10 mmol, 0.05 equiv), and DMF (75 mL) was heated for 16 h to give crude **16g** (1.29 g) as a black solid. DCM (5 mL) was added, and the precipitate was filtered and washed with DCM (2 × 5 mL) to give pure **16g** (255 mg) as a white solid. The filtrate was further purified by flash chromatography using a gradient of DCM/ethyl acetate as the eluting solvent to yield an additional **16g** (143 mg) for a combined yield of **16g** (398 mg, 47% yield). GC–MS shows one peak M^+^ = 423 (100%). ^1^H NMR (400 MHz; CDCl_3_): δ 8.27 (s, 1H), 7.73–7.71 (m, 2H), 7.66–7.64 (m, 2H), 7.49 (s, 1H), 7.35–7.33 (m, 2H), 7.05–7.03 (m, 2H), 4.09 (s, 3H), 3.90 (s, 3H), 2.53 (s, 3H).

#### 4,6-Dichloro-3-(4′-(difluoromethoxy)-[1,1′-biphenyl]-4-yl)-7-methoxy-2-methylquinoline (**16h**)

Following the general procedure for the preparation of biphenyl quinolines, a mixture of **14** (794 mg, 2.0 mmol, 1 equiv), **15h** (648 mg, 2.4 mmol, 1.2 equiv), K_2_CO_3_ (552 mg, 4.0 mmol, 2 equiv), Pd(dppf)Cl_2_ (73 mg, 0.10 mmol, 0.05 equiv), and DMF (75 mL) was heated for 16 h to give crude **16h** (1.073 g) as a black solid. DCM (5 mL) was added, and the precipitate was filtered, washed with DCM (2 × 5 mL), and then crystallized from DCM to give pure **16h** (412 mg) as a white solid. The filtrate was further purified by flash chromatography using a gradient of ethyl acetate/hexane as the eluting solvent to yield an additional **16h** (140 mg) for a combined yield of **16h** (552 mg, 60% yield). GC–MS shows one peak M^+^ = 459 (100%). ^1^H NMR (400 MHz; CDCl_3_): δ 8.27 (s, 1H), 7.74–7.68 (m, 4H), 7.49 (s, 1H), 7.39–7.36 (m, 2H), 7.28–7.24 (m, 2H), 6.60 (t, *J* = 73.8 Hz, 1H), 4.10 (s, 3H), 2.52 (d, *J* = 2.9 Hz, 3H).

#### 4,6-Dichloro-7-methoxy-2-methyl-3-(3′-(trifluoromethyl)-[1,1′-biphenyl]-4-yl)quinoline (**16i**)

Following the general procedure described by Nilsen et al.,^13^ a mixture of **14** (794 mg, 2.0 mmol, 1 equiv), **15i** (456 mg, 2.4 mmol, 1.2 equiv), K_2_CO_3_ (2 mL of 2 M solution, 552 mg, 4.0 mmol, 2 equiv), Pd(dppf)Cl_2_ (73 mg, 0.10 mmol, 0.05 equiv), and DMF (75 mL) was heated at 80 °C for 16 h to give crude **16i** (1.42 g) as a black solid. The product was soluble in DCM and was purified by flash chromatography using a gradient of ethyl acetate/hexane as the eluting solvent to give pure **16i** (445 mg, 48% yield) as a white solid. GC–MS shows one major peak M^+^ = 461 (100%). ^1^H NMR (400 MHz; CDCl_3_): δ 8.28 (s, 1H), 7.96–7.88 (m, 2H), 7.79–7.76 (m, 2H), 7.69–7.63 (m, 2H), 7.50 (s, 1H), 7.43–7.40 (m, 2H), 4.10 (s, 3H), 2.53 (s, 3H).

#### 4,6-Dichloro-7-methoxy-2-methyl-3-(3′-(trifluoromethoxy)-[1,1′-biphenyl]-4-yl)quinoline (**16j**)

Following the general procedure for the preparation of biphenyl quinolines, a mixture of **14** (794 mg, 2.0 mmol, 1 equiv), **15j** (494 mg, 2.4 mmol, 1.2 equiv), K_2_CO_3_ (552 mg, 4.0 mmol, 2 equiv), Pd(dppf)Cl_2_ (73 mg, 0.10 mmol, 0.05 equiv), and DMF (75 mL) was heated for 16 h to give crude **16j** (1.249 g) as a black solid. The product was soluble in DCM and was purified by flash chromatography using a gradient of ethyl acetate/hexane as the eluting solvent to give **16j** (639 mg, 67% yield) as a white solid. GC–MS shows one peak M^+^ = 477 (100%) and one minor peak M^+^ = 397 (100%) corresponding to the starting material **14**. GC–MS and NMR indicated that **16j** is pure (∼95%) enough to use for the next step. A sample was recrystallized in hexane/ethyl acetate to give pure **16j** as a white crystalline solid. ^1^H NMR (400 MHz; CDCl_3_): δ 8.28 (s, 1H), 7.75 (d, *J* = 7.7 Hz, 2H), 7.65–7.61 (m, 1H), 7.58–7.47 (m, 3H), 7.40 (d, *J* = 7.9 Hz, 2H), 7.29–7.26 (m, 2H, overlapping with residual of peak of CDCl_3_), 4.09 (s, 3H), 2.52 (s, 3H).

#### 4,6-Dichloro-7-methoxy-2-methyl-3-(4′-(4-(trifluoromethoxy)phenoxy)-[1,1′-biphenyl]-4-yl)quinoline (**16k**)

Following the general procedure for the preparation of biphenyl quinolines, a mixture of **14** (794 mg, 2.0 mmol, 1 equiv), **15k** (1.18 g, 3 mmol, 1.5 equiv), K_2_CO_3_ (552 mg, 4.0 mmol, 2 equiv), Pd(dppf)Cl_2_ (73 mg, 0.10 mmol, 0.05 equiv), and DMF (75 mL) was heated for 48 h to give crude **16k** (1.42 g) as a black solid. DCM (5 mL) was added, and the precipitate was filtered and washed with DCM (2 × 5 mL) to give pure **16k** (110 mg) as a white solid. The filtrate was further purified by flash chromatography using a gradient of ethyl acetate/hexane as the eluting solvent to yield additional **16k** (489 mg) for a combined yield of **16k** (599 mg, 53% yield). GC–MS shows one peak M^+^ = 569 (100%). ^1^H NMR (400 MHz; CDCl_3_): δ 8.28 (s, 1H), 7.75–7.72 (m, 2H), 7.71–7.69 (m, 2H), 7.50 (s, 1H), 7.39–7.35 (m, 2H), 7.26–7.23 (m, 2H), 7.17–7.13 (m, 2H), 7.12–7.08 (m, 2H), 4.10 (s, 3H), 2.53 (s, 3H).

#### 4,6-Dichloro-7-methoxy-2-methyl-3-(4-(4,4,5,5-tetramethyl-1,3,2-dioxaborolan-2-yl)phenyl)quinoline (**17**)

A mixture of **14** (7.08 g, 0.18 mol), bis(pinacolato)diboron (1.4 equiv, 6.34 g, 0.025 mol), and potassium acetate (3.0 equiv, 5.24 g, 0.0534 mol) in 250 mL *N*,*N*-dimethylformamide was degassed by bubbling argon through a glass tube inserted under the liquid surface for 20 min at room temperature. [1,1′-Bis(diphenylphosphino)ferrocene]-dichloropalladium(II) (5 mol %, 0.65 g, 0.00089 mol) was added followed by heating at 80 °C under an atmosphere of argon. After 72 h, TLC and GC–MS indicated that unreacted quinoline starting material was still present. The reaction was cooled to room temperature and again degassed, followed by the addition of further [1,1′-bis(diphenylphosphino)ferrocene]-dichloropalladium(II) (2.4 mol %, 0.32 g, 0.00044 mol). The reaction was again heated at 80 °C under an atmosphere of argon for 72 h. Although TLC and GC–MS showed that a small amount of unreacted 3-(4-bromophenyl)-4,6-dichloro-7-methoxy-2-methylquinoline still remained, the reaction was removed from the heat, filtered through Celite, and concentrated under reduced pressure with heating. The resulting black residue was taken up in dichloromethane (250 mL) and filtered through Celite. The dark filtrate was concentrated under reduced pressure with heating, affording a black sludge. This material was again taken up in dichloromethane (300 mL) and washed with 5% brine (2 × 100 mL) and then 10% brine (100 mL). The pooled organic layers were dried (MgSO_4_) and evaporated under reduced pressure with warming, affording a black solid (11.44 g). This material was taken up in 15 mL dichloromethane and filtered through a plug of silica gel (100 g, prewetted with dichloromethane), washing with 98/2 v/v dichloromethane/ethyl acetate until no more product eluted by TLC. Evaporation of the filtrate afforded a pale greenish gray solid (7.22 g). Automated flash chromatography on silica, eluting with a gradient of 100% dichloromethane to 98/2 v/v dichloromethane/ethyl acetate, afforded the desired product (*R*_f_ = 0.21, 98/2 v/v dichloromethane/ethyl acetate) as an off-white solid (3.66 g, 46%, ^1^H NMR (400 MHz; CDCl_3_): δ 8.23 (s, 1H), 7.97–7.94 (m, 2H), 7.46 (s, 1H), 7.30–7.27 (m, 2H), 4.06 (s, 3H), 2.43 (s, 3H), 1.38 (s, 12H)).

#### 4,6-Dichloro-7-methoxy-2-methyl-3-(3′-(pentafluorosulfanyl)-[1,1′-biphenyl]-4-yl)quinoline (**16l**)

A mixture of **17** (0.46 g, 0.0010 mol), anhydrous potassium carbonate (2.0 equiv, 0.0021 mol, 0.29 g), and *meta-*bromophenylsulfur pentafluoride (1.3 equiv, 0.0013 mol, 0.38 g) in *N*,*N*-dimethylformamide (55 mL) was degassed by bubbling argon through a glass tube under the liquid surface for 20 min at room temperature. [1,1′-Bis(diphenylphosphino)ferrocene]-dichloropalladium(II) (5 mol %, 0.038 g, 0.000052 mol) was added followed by heating at 80 °C under an atmosphere of argon for 22 h. The cooled reaction mixture was filtered through Celite. The filtrate was concentrated under reduced pressure with heating, and the resulting dark solid was taken up in dichloromethane (125 mL) and again filtered through Celite. The filtrate was adsorbed onto silica and purified by flash chromatography, eluting with a gradient of 95/5 to 77/23 v/v hexanes/ethyl acetate. The desired product (*R*_f_ = 0.41 (3/2 v/v hexanes/ethyl acetate, silica) was obtained as a white solid (0.45 g, 70%, ^1^H NMR (400 MHz; CDCl_3_): δ 8.26 (s, 1H), 8.06–8.04 (m, 1H), 7.83–7.81 (m, 1H), 7.78 (ddd, *J* = 8.3, 2.2, 1.0 Hz, 1H), 7.74–7.71 (m, 2H), 7.61–7.57 (m, 1H), 7.48 (s, 1H), 7.42–7.38 (m, 2H), 4.08 (s, 3H), 2.50 (s, 3H)).

#### 4,6-Dichloro-7-methoxy-2-methyl-3-(4′-(pentafluorosulfanyl)-[1,1′-biphenyl]-4-yl)quinoline (**16m**)

A mixture of **17** (0.46 g, 0.0010 mol), anhydrous potassium carbonate (2.0 equiv, 0.0021 mol, 0.29 g), and *para-*bromophenylsulfur pentafluoride (1.3 equiv, 0.0013 mol, 0.38 g) in *N*,*N*-dimethylformamide (75 mL) was degassed by bubbling argon through a glass tube under the liquid surface for 20 min at room temperature. [1,1′-Bis(diphenylphosphino)ferrocene]-dichloropalladium(II) (5 mol %, 0.038 g, 0.000052 mol) was added followed by heating at 80 °C under an atmosphere of argon for 22 h. The cooled reaction mixture was filtered through Celite. The filtrate was concentrated under reduced pressure with heating, and the resulting dark solid was taken up in dichloromethane (125 mL) and again filtered through Celite. The filtrate was adsorbed onto silica and purified by flash chromatography, eluting with a gradient of 95/5 to 75/25 v/v hexanes/ethyl acetate. This afforded the desired product (*R*_f_ = 0.43 (3/2 v/v hexanes/ethyl acetate, silica) as an off-white solid (0.38 g, 83%, ^1^H NMR (400 MHz; CDCl_3_): δ 8.25 (s, 1H), 7.89–7.86 (m, 2H), 7.77–7.72 (m, 4H), 7.48 (s, 1H), 7.41–7.38 (m, 2H), 4.08 (s, 3H), 2.50 (s, 3H)).

#### 6-Chloro-3-(4′-chloro-[1,1′-biphenyl]-4-yl)-7-methoxy-2-methylquinolin-4(1*H*)-one (**ELQ-637**)

Following the general procedure for the hydrolysis of 4-chloro quinolines, a mixture of **16a** (157 mg, 0.3 mmol, 1 equiv), KOAc (360 mg, 3.7 mmol, 10 equiv), and glacial acetic acid (10 mL) was heated for 26 h. After cooling to room temperature, the reaction mixture was further cooled to 4 °C. The resulting precipitate was filtered, washed with excess water followed by acetone (3 × 5 mL), and air-dried to give pure **ELQ-637** as a pale taupe powder (0.104 g, yield 69%). ^1^H NMR (400 MHz; DMSO-*d*_6_): δ 11.70 (s, 1H), 8.01 (s, 1H), 7.78–7.73 (m, 2H), 7.72–7.66 (m, 2H), 7.57–7.51 (m, 2H), 7.38–7.32 (m, 2H), 7.08 (s, 1H), 3.97 (s, 3H), 2.26 (s, 3H); mp 376.9–378.4 °C (decomposed). HRMS calculated for C_23_H_18_Cl_2_N_1_O_2_ [M + H]^+^ = 410.0709, observed for [M + H]^+^ = 410.0703.

#### 6-Chloro-7-methoxy-2-methyl-3-(4′-methyl-[1,1′-biphenyl]-4-yl)quinolin-4(1*H*)-one (**ELQ-603**)

Following the general procedure for the hydrolysis of 4-chloro quinolines, a mixture of **16b** (204 mg, 0.5 mmol, 1 equiv), KOAc (490 mg, 5.0 mmol, 10 equiv), and glacial acetic acid (5 mL) was heated for 16 h. After cooling to room temperature, the reaction mixture was poured into ice water (20 mL). The resulting precipitate was filtered; washed with water (3 × 10 mL), acetone (2 × 10 mL), DCM (2 × 10 mL), and hexane (10 mL); and air-dried to give pure **ELQ-603** (170 mg, 87% yield) as a white solid. ^1^H NMR (400 MHz; DMSO-*d*_6_): δ 11.69–11.67 (s, 1H), 8.02 (s, 1H), 7.66–7.60 (m, 4H), 7.32–7.28 (m, 4H), 7.08 (s, 1H), 3.97 (s, 3H), 2.36–2.33 (s, 3H), 2.25 (s, 3H); mp 384.9–386.0 °C (decomposed). HRMS calculated for C_24_H_21_Cl_1_N_1_O_2_ [M + H]^+^ = 390.1255, observed for [M + H]^+^ = 390.1246.

#### 3-(4′-(*tert*-Butyl)-[1,1′-biphenyl]-4-yl)-6-chloro-7-methoxy-2-methylquinolin-4(1*H*)-one (**ELQ-651**)

Following the general procedure for the hydrolysis of 4-chloro quinolines, a mixture of **16c** (225 mg, 0.5 mmol, 1 equiv), KOAc (490 mg, 5.0 mmol, 10 equiv), and glacial acetic acid (5 mL) was heated for 16 h. After cooling to room temperature, the reaction mixture was poured into ice water (20 mL). The resulting precipitate was filtered; washed with water (3 × 10 mL), acetone (2 × 10 mL), DCM (2 × 10 mL), and hexane (10 mL); and air-dried to give pure **ELQ-651** (184 mg, 85% yield) as a white solid. ^1^H NMR (400 MHz; DMSO-*d*_6_): δ 11.68 (s, 1H), 8.02 (s, 1H), 7.67–7.64 (m, 4H), 7.51 (m, 2H), 7.34–7.32 (m, 2H), 7.09 (s, 1H), 3.98 (s, 3H), 2.27 (s, 3H), 1.34 (s, 9H); mp > 400 °C with some decomposition observed above 390 °C. HRMS calculated for C_27_H_27_Cl_1_N_1_O_2_ [M + H]^+^ = 432.1725, observed for [M + H]^+^ = 432.1717.

#### 6-Chloro-7-methoxy-2-methyl-3-(4′-(trifluoromethyl)-[1,1′-biphenyl]-4-yl)quinolin-4(1*H*)-one (**ELQ-647**)

Following the general procedure for the hydrolysis of 4-chloro quinolines, a mixture of **16d** (231 mg, 0.5 mmol, 1 equiv), KOAc (490 mg, 5.0 mmol, 10 equiv), and glacial acetic acid (5 mL) was heated for 16 h. After cooling to room temperature, the reaction mixture was poured into ice water (20 mL). The resulting precipitate was filtered; washed with water (3 × 10 mL), acetone (2 × 10 mL), DCM (2 × 10 mL), and hexane (10 mL); and air-dried to give pure **ELQ-647** (180 mg, 81% yield) as a white solid. ^1^H NMR (400 MHz; DMSO-*d*_6_): δ 11.71 (s, *J* = 0.2 Hz, 1H), 8.02 (s, *J* = 1.7 Hz, 1H), 7.98–7.95 (m, 2H), 7.85–7.83 (m, 2H), 7.79–7.77 (m, 2H), 7.41–7.39 (m, 2H), 7.10 (s, 1H), 3.98 (s, 3H), 2.27 (s, 3H); mp 381.0–381.8 °C (decomposed). HRMS calculated for C_24_H_18_Cl_1_F_3_N_1_O_2_ [M + H]^+^ = 444.0973, observed for [M + H]^+^ = 444.0962.

#### 4′-(6-Chloro-7-methoxy-2-methyl-4-oxo-1,4-dihydroquinolin-3-yl)-[1,1′-biphenyl]-4-carbonitrile (**ELQ-602**)

Following the general procedure for the hydrolysis of 4-chloro quinolines, a mixture of **16e** (231 mg, 0.5 mmol, 1 equiv), KOAc (490 mg, 5.0 mmol, 10 equiv), and glacial acetic acid (5 mL) was heated for 16 h. After cooling to room temperature, the reaction mixture was poured into ice water (20 mL). The resulting precipitate was filtered; washed with water (3 × 10 mL), acetone (2 × 10 mL), DCM (2 × 10 mL), and hexane (10 mL); and air-dried to give pure **ELQ-602** (172 mg, 86% yield) as a white solid. ^1^H NMR (400 MHz; DMSO-*d*_6_): δ 11.70 (s, 1H), 7.96 (broad d, *J* = 32.4 Hz, 4H), 7.77 (broad d, *J* = 7.9 Hz, 2H), 7.39 (broad d, *J* = 7.8 Hz, 2H), 7.07 (s, 1H), 3.97 (s, 3H), 2.26 (s, 3H); mp 354.0–355.5 °C (decomposed). HRMS calculated for C_24_H_18_Cl_1_N_2_O_2_ [M + H]^+^ = 401.1051, observed for [M + H]^+^ = 401.1044.

#### 6-Chloro-3-(4′-(difluoromethyl)-[1,1′-biphenyl]-4-yl)-7-methoxy-2-methylquinolin-4(1*H*)-one (**ELQ-659**)

Following the general procedure for the hydrolysis of 4-chloro quinolines, a mixture of **16f** (222 mg, 0.5 mmol, 1 equiv), KOAc (490 mg, 5.0 mmol, 10 equiv), and glacial acetic acid (5 mL) was heated for 16 h. After cooling to room temperature, the reaction mixture was poured into ice water (20 mL). The resulting precipitate was filtered; washed with water (3 × 10 mL), acetone (2 × 10 mL), methylene chloride (2 × 10 mL), and hexane (10 mL); and air-dried to give pure **ELQ-659** (151 mg, 71% yield) as a white solid. ^1^H NMR (400 MHz; DMSO-*d*_6_): δ 11.70 (s, 1H), 8.02 (s, 1H), 7.87 (d, *J* = 7.9 Hz, 2H), 7.74 (d, *J* = 7.9 Hz, 2H), 7.68 (d, *J* = 7.9 Hz, 2H), 7.37 (d, *J* = 7.9 Hz, 2H), 7.10 (t, *J* = 56.3 Hz, 1H), 7.09 (s, 1H), 3.98 (s, 3H), 2.27 (s, 3H); mp 345.8–346.7 °C (decomposed). HRMS calculated for C_24_H_19_Cl_1_F_2_N_1_O_2_ [M + H]^+^ = 426.1067, observed for [M + H]^+^ = 426.1059.

#### 6-Chloro-7-methoxy-3-(4′-methoxy-[1,1′-biphenyl]-4-yl)-2-methylquinolin-4(1*H*)-one (**ELQ-645**)

Following the general procedure for the hydrolysis of 4-chloro quinolines, a mixture of **16g** (212 mg, 0.5 mmol, 1 equiv), KOAc (490 mg, 5.0 mmol, 10 equiv), and glacial acetic acid (5 mL) was heated for 16 h. After cooling to room temperature, the reaction mixture was poured into ice water (20 mL). The resulting precipitate was filtered; washed with water (3 × 10 mL), acetone (2 × 10 mL), DCM (2 × 10 mL), and hexane (10 mL); and air-dried to give pure **ELQ-645** (160 mg, 79% yield) as a white solid. ^1^H NMR (400 MHz; DMSO-*d*_6_): δ 11.67 (s, 1H), 8.02 (s, 1H), 7.68–7.62 (m, 3H), 7.32–7.29 (m, 2H), 7.09–7.04 (m, 2H), 3.98 (s, 3H), 3.82 (s, 3H), 2.26 (s, 2H); mp 364.1–365.0 °C (decomposed). HRMS calculated for C_24_H_21_Cl_1_N_1_O_3_ [M + H]^+^ = 406.1204, observed for [M + H]^+^ = 406.1197.

#### 6-Chloro-3-(4′-(difluoromethoxy)-[1,1′-biphenyl]-4-yl)-7-methoxy-2-methylquinolin-4(1*H*)-one (**ELQ-600**)

Following the general procedure for the hydrolysis of 4-chloro quinolines, a mixture of **16h** (230 mg, 0.5 mmol, 1 equiv), KOAc (490 mg, 5.0 mmol, 10 equiv), and glacial acetic acid (5 mL) was heated for 16 h. After cooling to room temperature, the reaction mixture was poured into ice water (20 mL). The resulting precipitate was filtered; washed with water (3 × 10 mL), acetone (2 × 10 mL), DCM (2 × 10 mL), and hexane (10 mL); and air-dried to give pure **ELQ-600** (165 mg, 75% yield) as a white solid. ^1^H NMR (400 MHz; DMSO-*d*_6_): δ 11.74 (s, 1H), 8.02 (s, 1H), 7.77 (broad d, *J* = 8.7 Hz, 2H), 7.67 (broad d, *J* = 8.3 Hz, 2H), 7.31 (t, *J* = 73.8 Hz, 1H)), 7.34 (broad d, *J* = 8.3 Hz, 2H), 7.29 (broad d, *J* = 8.6 Hz, 2H), 7.07 (s, 1H), 3.96 (s, 3H), 2.25 (s, 3H); mp 336.1–336.9 °C (decomposed). HRMS calculated for C_24_H_19_Cl_1_F_2_N_1_O_3_ [M + H]^+^ = 442.1016, observed for [M + H]^+^ = 442.1009.

#### 6-Chloro-7-methoxy-2-methyl-3-(3′-(trifluoromethyl)-[1,1′-biphenyl]-4-yl)quinolin-4(1*H*)-one (**ELQ-646**)

Following the general procedure for the hydrolysis of 4-chloro quinolines, a mixture of **16i** (231 mg, 0.5 mmol, 1 equiv), KOAc (490 mg, 5.0 mmol, 10 equiv), and glacial acetic acid (5 mL) was heated for 16 h. After cooling to room temperature, the reaction mixture was poured into ice water (20 mL). The resulting precipitate was filtered; washed with water (3 × 10 mL), acetone (2 × 10 mL), DCM (2 × 10 mL), and hexane (10 mL); and air-dried to give **ELQ-646** (177 mg, 80% yield) as a white solid. The product is 95–98% by ^1^H NMR and HPLC. ^1^H NMR (400 MHz; DMSO-*d*_6_): δ 11.70 (s, 1H), 8.11–7.99 (m, 3H), 7.85–7.70 (m, 4H), 7.44–7.35 (m, 2H), 7.10 (s, 1H), 3.97 (s, 3H), 2.27 (s, 3H); mp 338.8–340.4 °C (decomposed). HRMS calculated for C_24_H_18_Cl_1_F_3_N_1_O_2_ [M + H]^+^ = 444.0973, observed for [M + H]^+^ = 444.0965.

#### 6-Chloro-7-methoxy-2-methyl-3-(3′-(trifluoromethoxy)-[1,1′-biphenyl]-4-yl)quinolin-4(1*H*)-one (**ELQ-604**)

Following the general procedure for the hydrolysis of 4-chloro quinolines, a mixture of **16j** (476 mg, 1.0 mmol, 1 equiv), KOAc (980 mg, 10.0 mmol, 10 equiv), and glacial acetic acid (5 mL) was heated for 16 h. After cooling to room temperature, the reaction mixture was poured into ice water (20 mL). The resulting precipitate was filtered; washed with water (3 × 10 mL), acetone (2 × 10 mL), DCM (2 × 10 mL), and hexane (10 mL); and air-dried to give crude **ELQ-604** (350 mg). The product was crystallized from DMF to give **ELQ-604** (200 mg, 43% yield). ^1^H NMR and HPLC indicated that the product was about 95–98% pure. ^1^H NMR (400 MHz; DMSO-*d*_6_): δ 11.70 (s, 1H), 8.02 (s, 1H), 7.79 (ddd, *J* = 7.9, 1.7, 0.9 Hz, 1H), 7.76–7.73 (m, 2H), 7.69 (s, 1H), 7.63 (t, *J* = 8.0 Hz, 1H), 7.40–7.36 (m, 3H), 7.09 (s, 1H), 3.98 (s, 3H), 2.27 (s, 3H); mp 325.7–327.2 °C (decomposed). HRMS calculated for C_24_H_18_Cl_1_F_3_N_1_O_3_ [M + H]^+^ = 460.0922, observed for [M + H]^+^ = 460.0915.

#### 6-Chloro-7-methoxy-2-methyl-3-(4′-(4-(trifluoromethoxy)phenoxy)-[1,1′-biphenyl]-4-yl)quinolin-4(1*H*)-one (**ELQ-653**)

Following the general procedure for the hydrolysis of 4-chloro quinolines, a mixture of **16k** (285 mg, 0.5 mmol, 1 equiv), KOAc (490 mg, 5.0 mmol, 10 equiv), and glacial acetic acid (5 mL) was heated for 16 h. After cooling to room temperature, the reaction mixture was poured into ice water (20 mL). The resulting precipitate was filtered; washed with water (3 × 10 mL), acetone (2 × 10 mL), methylene chloride (2 × 10 mL), and hexane (10 mL); and air-dried to give pure **ELQ-653** (213 mg, 77% yield) as a white solid. ^1^H NMR and HPLC indicated that the product was about 95–98% pure. ^1^H NMR (400 MHz; DMSO-*d*_6_): δ 11.73–11.69 (s, 1H), 8.02 (s, 1H), 7.78–7.76 (m, 2H), 7.69–7.67 (m, 2H), 7.44–7.41 (m, 2H), 7.35–7.33 (m, 2H), 7.21–7.16 (m, 4H), 7.09 (s, 1H), 3.98 (s, 3H), 2.27 (s, 3H); mp 332.6–335.0 °C (decomposed). HRMS calculated for C_30_H_22_Cl_1_F_3_N_1_O_4_ [M + H]^+^ = 552.1184, observed for [M + H]^+^ = 552.1176.

#### 6-Chloro-7-methoxy-2-methyl-3-(3′-(pentafluorosulfanyl)-[1,1′-biphenyl]-4-yl)quinolin-4(1*H*)-one (**ELQ-662**)

4,6-Dichloro-7-methoxy-2-methyl-3-(3′-(pentafluorosulfanyl)-[1,1′-biphenyl]-4-yl)quinoline (0.45 g, 0.00086 mol) and potassium acetate (10 equiv, 0.0086 mol, 0.85 g) were heated in glacial acetic acid (9 mL) at 120 °C for 6 h. After cooling, the reaction mixture was chilled at 5 °C for 30 min. Vacuum filtration and rinsing with excess water followed by acetone (5 × 1.5 mL) afforded the desired product as fine white crystals (0.21 g, 48%, ^1^H NMR (400 MHz; DMSO-*d*_6_): δ 11.75 (s, 1H), 8.14–8.11 (m, 1H), 8.05–8.02 (m, 2H), 7.92 (ddd, *J* = 8.3, 2.3, 0.8 Hz, 1H), 7.77–7.72 (m, 3H), 7.41–7.38 (m, 2H), 7.09 (s, 1H), 3.97 (s, 3H), 2.26 (s, 3H)); HPLC shows purity >98%, mp 352.4–352.8 °C (decomposed). HRMS calculated for C_23_H_18_Cl_1_F_5_N_1_O_2_S_1_ [M + H]^+^ = 502.0661, observed for [M + H]^+^ = 502.0654.

#### 6-Chloro-7-methoxy-2-methyl-3-(4′-(pentafluorosulfanyl)-[1,1′-biphenyl]-4-yl)quinolin-4(1*H*)-one (**ELQ-663**)

4,6-Dichloro-7-methoxy-2-methyl-3-(4′-(pentafluorosulfanyl)-[1,1′-biphenyl]-4-yl)quinoline (0.38 g, 0.00073 mol) and potassium acetate (10 equiv, 0.0073 mol, 0.72 g) were heated in glacial acetic acid (12 mL) at 120 °C for 6 h. After cooling, the reaction mixture was chilled at 5 °C for 30 min. Vacuum filtration and rinsing with excess water followed by acetone (5 × 1.5 mL) afforded the desired product as silver crystals (0.26 g, 72%, ^1^H NMR (400 MHz; DMSO-*d*_6_): δ 11.71 (s, 1H), 8.02 (s, 1H), 8.01–7.98 (m, 2H), 7.97–7.93 (m, 2H), 7.78–7.75 (m, 2H), 7.42–7.39 (m, 2H), 7.09 (s, 1H), 3.97 (s, 3H), 2.27 (s, 3H)); HPLC shows purity >98%, mp 373.1–375.0 °C (decomposed). HRMS calculated for C_23_H_18_Cl_1_F_5_N_1_O_2_S_1_ [M + H]^+^ = 502.0661, observed for [M + H]^+^ = 502.0655.

#### (((6-Chloro-7-methoxy-2-methyl-3-(4′-(trifluoromethoxy)-[1,1′-biphenyl]-4-yl)quinolin-4-yl)oxy)methyl ethyl carbonate (**ELQ-598**)

A stirred mixture of **ELQ-596** (460 mg, 1.0 mmol, 1 equiv), tetra-butyl ammonium iodide (742 mg, 2.0 mmol, 2 equiv), chloromethyl ethylcarbonate (278 mg, 2.0 mmol, 2 equiv), and dry K_2_CO_3_ (278 mg, 2.0 mmol, 2 equiv) in DMF (25 mL) was heated at 60 °C for 24 h, whereupon TLC showed that no more starting material remained. The mixture was cooled to room temperature and filtered, and the filtrate was concentrated to dryness to give 700 mg of a brown oil. The resulting residue was stirred with ethyl acetate (50 mL) for 30 min, and the insoluble tetra-butyl ammonium iodide was removed by vacuum filtration and washing with ethyl acetate (3 × 10 mL). The filtrate was concentrated to dryness and purified by flash chromatography using a gradient of ethyl acetate/hexane as eluent to give pure **ELQ-598** (412 mg, 73% yield) as a white solid. HPLC shows purity greater than 98%. GC–MS shows one peak M^+^ = 561 (53%), 459 (100%). ^1^H NMR (400 MHz; CDCl_3_): δ 8.08 (s, 1H), 7.74–7.70 (m, 4H), 7.51–7.48 (m, 2H), 7.46 (s, 1H), 7.37–7.34 (m, 2H), 5.31 (s, 2H), 4.13 (q, *J* = 7.1 Hz, 2H), 4.07 (s, 3H), 2.56 (s, 3H), 1.22 (t, *J* = 7.1 Hz, 3H); mp 139.7–140.1 °C. HRMS calculated for C_28_H_24_Cl_1_F_3_N_1_O_6_ [M + H]^+^ = 562.1239, observed for [M + H]^+^ = 562.1227.

#### X-ray Crystallography of **ELQ-331** and **ELQ-598**

##### X-ray Crystallography

Diffraction intensities for **ELQ-331** and **ELQ-598** were collected at 173 K on a Bruker Apex2 DUO CCD diffractometer using CuKα radiation, λ = 1.54178 Å. Absorption correction was applied by SADABS.^28^ Space group was determined based on intensity statistics. Structure was solved by direct methods and Fourier techniques and refined on *F*^2^ using full matrix least-squares procedures. All non-H atoms were refined with anisotropic thermal parameters. Positions of H atoms were found on the residual density map and refined with isotropic thermal parameters. In the crystal structure of **ELQ-598**, there are two symmetrically independent conformers labeled as **ELQ-598A** and **ELQ-598B**. The molecules in the crystal structure are joined via a network of H-bonds. All calculations were performed by the Bruker SHELXL-2014/7 package.^29^

##### Crystallographic Data for **ELQ-331**

C_28_H_23_ClF_3_NO_7_, *M* = 577.92, 0.17 × 0.14 × 0.12 mm, *T* = 173(2) K, triclinic, space group *P-*1, *a* = 8.2660(4) Å, *b* = 12.2303(6) Å, *c* = 13.8274(7) Å, α = 98.357(2)°, β = 106.128(2)°, γ = 98.365(3)°, *V* = 1302.97(11) Å^3^, *Z* = 2, *D*_c_ = 1.473 Mg/m^3^, μ(Cu) = 1.929 mm^–1^, *F*(000) = 596, 2θ_max_ = 133.27°, 12072 reflections, 4581 independent reflections [*R*_int_ = 0.0437], R1 = 0.0479, wR2 = 0.1294, and GOF = 1.050 for 4581 reflections (453 parameters) with *I* > 2σ(*I*), R1 = 0.0526, wR2 = 0.1345, and GOF = 1.050 for all reflections, max/min residual electron density +0.936/–0.511 eÅ^–3^.

##### Crystallographic Data for **ELQ-598**

C_28_H_23_ClF_3_NO_6_, *M* = 561.92, 0.14 × 0.10 × 0.06 mm, *T* = 173(2) K, triclinic, space group *P*-1, *a* = 7.8715(2) Å, *b* = 12.9032(4) Å, *c* = 25.1245(7) Å, α = 93.716(2)°, β = 90.800(2)°, γ = 92.125(2)°, *V* = 2544.37(12) Å^3^, *Z* = 4, *D*_c_ = 1.467 Mg/m^3^, μ(Cu) = 1.928 mm^–1^, *F*(000) = 1160, 2θ_max_ = 133.77°, 28338 reflections, 8959 independent reflections [*R*_int_ = 0.0397], R1 = 0.0522, wR2 = 0.1433, and GOF = 1.035 for 8959 reflections (887 parameters) with *I* > 2σ(*I*), R1 = 0.0588, wR2 = 0.1489, and GOF = 1.035 for all reflections, max/min residual electron density +0.837/–0.372 eÅ^–3^.

#### In Vitro Metabolic Stability Assay

Metabolic stability studies of **ELQ-596** were performed at ChemPartner, Shanghai, China. The drug was incubated at 37 °C and 1 μM concentration in murine liver microsomes (Corning) for 45 min at a protein concentration of 0.5 mg/mL in potassium phosphate buffer at pH 7.4 containing 1.0 mM EDTA. The metabolic reaction was initiated by addition of NADPH and quenched with ice-cold acetonitrile at 0, 5, 15, 25, and 45 min. The progress of compound metabolism was followed by LC–MS/MS (ESI positive ion, LC–MS/MS-034 (API-6500+) using a C_18_ stationary phase (ACQUITY UPLC BEH C_18_ (2.1 Å, ∼50 mm, 1.7 μm)) and a MeOH/water mobile phase containing 0.25% FA and 1 mM NH4OAc. Imipramine or osalmid was used as internal standard, and ketanserin was used as a control drug with intermediate stability. Concentration versus time data for each compound were fitted to an exponential decay function to determine the first-order rate constant for substrate depletion, which was then used to calculate the degradation half-life (*t*_1/2_) and predicted intrinsic clearance value (Cl_int_) from an assumed murine hepatic blood flow of 90 mL/min/kg.

#### *Plasmodium falciparum* Culture

Laboratory strains of *P. falciparum* were cultured in human erythrocytes by standard methods. The parasites were grown in culture medium with fresh human erythrocytes maintained at 2% hematocrit at 37 °C in low-oxygen conditions (5% O_2_, 5% CO_2_, 90% N_2_). The culture medium was RPMI 1640 supplemented with 25 mM HEPES buffer, gentamicin sulfate (25 mg/Liter), hypoxanthine (45 mg/Liter), 10 mM glucose, 2 mM glutamine, and 0.5% Albumax II (complete medium). Cultures were maintained at less than 10% parasitemia by transfer of infected cells to fresh erythrocytes and culture medium every 3 or 4 days. The *P. falciparum* strains used in these experiments include the following: D6 (MRA-285/BEI Resources, deposited by Dr. Dennis Kyle) has modest resistance to mefloquine but is generally drug sensitive; Dd2 (MRA-150/BEI Resources, deposited by Dr. David Walliker) has resistance to chloroquine, mefloquine, and pyrimethamine; D1 is a subclone of Dd2 that was selected for resistance to **ELQ-300**; and Tm90-C2B was isolated from a patient enrolled in an atovaquone clinical trial in Thailand upon recrudescence after cessation of drug treatment (obtained from Drs. Dennis Kyle and Victor Melendez, WRAIR).

#### In Vitro Drug Susceptibility Assays

SYBR green I assay. In vitro antiplasmodial activity was assessed using a published SYBR Green I fluorescence-based method.^20^ The drugs were added to 96-well plates using twofold serial dilutions in complete medium. The initial range was from 250 to 0.25 nM. Asynchronous *P. falciparum* parasites were diluted with uninfected erythrocytes and added to the wells to give a final culture volume of 100 μL at 2% hematocrit and 0.2% parasitemia. The plates were incubated for 72 h at 37 °C. The parasites were then lysed by adding 100 μL of SYBR green I lysis buffer containing 0.2 μL/ml SYBR green I dye (10,000×) in 20 mM Tris (pH 7.5), 5 mM EDTA, 0.008% (wt/vol) saponin, and 0.08% (v/v) Triton X-100. The plates were incubated at room temperature for an hour in the dark. The fluorescence signal, correlating to parasite DNA, was measured using a SpectraMax iD3 iD5Multi-Mode Microplate Reader, with excitation and emission wavelength bands centered at 497 and 520 nm, respectively. The 50% inhibitory concentrations (IC_50_'s) were determined by nonlinear regression analysis using the GraphPad Prism software. Drugs were assayed in quadruplicate, and the results were averaged during analysis to give a final IC_50_ value together with 95% confidence intervals. Atovaquone and **ELQ-300** were used as internal controls to verify cross-resistance and parasite strain integrity. If the IC_50_ value fell outside of the initially tested range, then the range was adjusted up or down, and the assay was repeated twice.

### In Vivo Efficacy against Murine Malaria

All animal experiments described in this report have undergone ethical review and approval by the institutional IACUC committee of the Portland VA Medical Center (VAMC) where the studies took place. All studies were carried out in accordance with the Portland VAMC’s policies regarding the Care, Welfare, and Treatment of animals.

The *P. yoelii* 4 day test monitors suppression of patent infection in female CF1 mice. The test began with the inoculation (iv) of parasitized erythrocytes (3.5 × 10^4^*P. yoelli*) (from a donor animal) on the first day of the experiment (D0). After 24 h, test drugs (including **ELQ-596** and prodrug **ELQ-598**) were administered daily by gavage for 4 successive days. Initially, the 3-biaryl-ELQs were tested at doses of 0.0025, 0.005, 0.01, 0.03, 0.1, 0.3, 1.0, and 10 mg/kg/day, including a vehicle-only (PEG400) control. After completion of drug treatment, a blood sample was collected (by pricking the tail vein) for determination of parasite burden beginning on the day after the final dose (D5). Percent parasitemia was assessed by direct microscopic analysis of Giemsa-stained blood smears. Drug activity was recorded as % suppression of parasite burden relative to drug-free controls. Animals with observable parasitemia following the experiment were euthanized; animals cleared of parasites from the bloodstream were observed daily with assessment of parasitemia performed weekly until day 30, at which point the animals were scored as cured of infection. Typically, the percentage parasitemia in untreated control animals on day 5 of the “4 day test” is between 20 and 25%. Nonlinear regression analysis was used for objective determination of ED_50_’s and ED_90_’s from the accumulated data as well as the nonrecrudescence dose (NRD). The 4 day test protocol was reviewed and approved by the local IACUC board at the Portland VA Medical Center. Experiments were performed with four mice per group to ensure statistical accuracy. Control drugs for these experiments included **ELQ-300** and prodrug **ELQ-331**.

#### In Vivo Single-Dose Efficacy against Murine Malaria

The effectiveness of selected 3-biaryl ELQs and prodrugs was assessed vs the blood-stage infection for single-dose cures. Mice were infected iv with 3.5 × 10^4^*P. yoelii* infected RBCs as described for the 4 day test above. Drug administration occurred on the day after inoculation (day 1). Test agents were dissolved in PEG-400 and administered ig once. On the fifth day, blood films were prepared, and % parasitemia was assessed. Animals remaining parasite-free for 30 days after drug administration were considered cured. The initial dosing range was 0.25, 0.5, 1, 3, and 10 mg/kg, including a control. Experiments were performed with four mice per group to ensure statistical accuracy. The reported parameter for these studies is the lowest single dose that provides a cure to all four animals in the group. **ELQ-331** served as a positive control in these studies to directly compare with prodrug **ELQ-598**.

#### In Vivo Prophylaxis against Sporozoite-induced Murine Malaria

**ELQ-598** was evaluated for causal prophylactic liver-stage activity in vivo in a *P. yoelii* sporozoite-challenge model at the Portland VA Medical Center. Methods were similar to those described previously by Zhang et al. in 2005.^30^ In brief, *P. yoelii* sporozoites (17XNL luciferase and green fluorescent protein transfected strain) were reared in *Anopheles stephensii* at the OHSU insectary (Dr. Brandon Wilder). Mice were inoculated with 10,000 sporozoites via tail vein injection of CF1 mice treated with or without drug (dissolved in PEG400) 1 h after inoculation. Blood samples were collected to monitor for the passage of parasites from the liver to the bloodstream at 72 and 120 h postinoculation and then weekly thereafter. Additional monitoring for blood-stage infection was continued for a 30 day period to confirm true causal prophylaxis against *P. yoelii* challenge. Untreated controls were included to establish the infectivity of sporozoites, and studies also included an internal positive control with **ELQ-331**. Testing involved the use of four animals per group for statistical accuracy. **ELQ-331** was used as a positive control.^10^ A minimum of 5000 red blood cells were examined to establish blood-stage infection. Blood smears were fixed with methanol, stained with Giemsa, and viewed under oil immersion at 1000× magnification under standard light microscopy. It is noteworthy that because of the relatively short pre-erythrocytic liver stage of *P. yoelii* (∼48 h), this test is considered to be a test for “presumptive causal prophylaxis” and requires confirmation by additional testing.

#### Production of *P. yoelii* Sporozoites

*Anopheles stephensi* mosquitoes were reared in the insectary at the Oregon Health and Science University, Vaccine and Gene Therapy Institute as described previously.^31^ Mosquitoes were infected with *Plasmodium yoelii* 17XNL strain modified to express green fluorescent protein (GFP) and luciferase^32^ as follows: female Swiss Webster mice (Charles River Laboratories) were injected intraperitoneally with blood-stage parasites, and gametocyte exflagellation was confirmed 2–3 days later. Infected mice were used to feed 3–7-day old female *Anopheles stephensi* mosquitoes by placing anesthetized mice on the mosquito cage to allow for feeding over 30 min. Mosquitoes were maintained at 26 °C and 80% humidity and provided with water and sucrose until dissection. Ten days after infection, a small number of mosquitoes were dissected to confirm the presence of oocysts in the mosquito midgut. Mosquitoes were dissected 14–16 days after infection, and salivary glands were collected.^33^ Sporozoites were collected from salivary glands by manual disruption and low-speed centrifugation and quantified for cryopreservation according to published protocol.^34^ Briefly, the sporozoites were pelleted by centrifugation and resuspended in RPMI 1640 (Cytiva HyCloneTM) to a volume 25% of the final freezing volume. The remaining 75% volume of CryoStor CS2 (Biolife Solutions) was added, and the sporozoites were aliquoted into cryovials. Cryovials were maintained at 4 °C for 30 min and then moved to −80 °C for 60 min before long-term storage in vapor-phase liquid nitrogen until use for mouse infections.

#### Processing of Liver Tissue for Assessment of Human Cytochrome *bc*_1_

All use of human tissue was approved by the OHSU IRB. Approximately 10 g of human liver tissue was obtained intraoperatively and placed in ice-cold PBS containing 5 mM glucose. [Note: The specimen was obtained from grossly normal tissue that was resected around the margins of a tumor as part of the standard surgical treatment for hepatic tumors.] Liver was homogenized using a razor blade followed by a Dounce homogenizer. HALT Protease Inhibitor Cocktail (Pierce) was added per the manufacturer’s instructions. A crude mitochondrial fraction was prepared by differential centrifugation as described in Renault et al.^35^ The final pellet was resuspended in storage buffer (50 mM tricine, 100 mM KCl, 2 mM NaN, 2.0% dodecyl-beta-d-maltoside, 30% glycerol, pH 8.0). Aliquots of this mitochondrial preparation were frozen in a dry ice–ethanol bath and stored at −80 °C until use.

#### Human Cytochrome *bc*_1_ Assays

Two microliters of mitochondrial preparation was resuspended in the assay buffer (50 mM tricine, 100 mM KCl, 2 mM NaN_3_, 0.1% dodecyl-beta-d-maltoside). **ELQ-596** was dissolved in DMSO as a 1 mM stock. Antimycin A was dissolved in ethanol as a 1 mM stock. Decylubiquinone was reduced to decylubiquinol with an excess of NaBH_4_, and the excess NaBH_4_ was subsequently quenched with hydrochloric acid. Cytochrome *c* (horse heart, Sigma) was added to 50 μM to start the reaction. The absorbance at 542 and 550 nm was measured every 0.5 s for 30 s in a BMG SPECTOstar Omega plate reader (BMG Labtech, Ortenberg, Germany). The rate of the nonenzymatic reaction of cytochrome *c* and decylubiquinol was measured separately in the absence of mitochondrial preparation and found to be insignificant. The concentrations of **ELQ-596** and antimycin A ranged from 156 to 10,000 nM. The reaction rates (*A*_550_ – *A*_542_ versus time) were plotted against log(concentration) and fit to a four-parameter logistic regression to estimate values of EC_50_ using Prism 9.0 for Mac OS (GraphPad Software, San Diego, California).

#### Mitochondrial Toxicity of 3-Biaryl ELQs

HepG2 was routinely cultured in DMEM containing 10% fetal calf serum and 100 IU/mL penicillin and 100 μg/mL streptomycin (complete medium). For studies to investigate the potential for mitochondrial toxicity by selected 3-biaryl-ELQs, the medium contained galactose rather than glucose to force the cells to produce ATP through oxidative phosphorylation, which in turn makes them sensitive to mitochondrial toxicants.^36^ Five thousand cells were added to each well of a 96-well plate with complete medium, and the cells were incubated for 72 h at 37 °C in a humidified atmosphere of 95% humidity and 5% CO_2_. After this period, the culture medium was removed, and the cells were shifted to galactose-containing medium with test drugs at concentrations ranging from 0 to 10 μM. Drug dilutions were prepared from DMSO stocks into galactose-containing medium. All plates were incubated for 24 h at 37 °C. After incubation, the contents of each well were removed, and 25 μL of TiterGlo II reagent mix containing luciferase, detergent, and luciferin was added to each well along with 25 μL of culture medium. The luminescence signal was allowed to develop and stabilize at room temperature for 15 min. At this point, the plates were read on a luminometer (SpectraMax iD3 iD5Multi-Mode Microplate Reader), and the data were processed to yield the percent decrease in ATP production relative to no-drug control values. These values reflect the potential damage caused by mitochondrial toxicants due to reduced intracellular ATP levels caused by interference with the human mitochondrial electron transport chain. Assays were set up in triplicate. Rotenone was used as a positive control for mitochondrial toxicity, whereas **ELQ-300** was used as a negative control.

##### Drug Administration and Pharmacokinetic Analysis

The PK experiments and quantifications of **ELQ-598** and **ELQ-596** were performed by ChemPartner Co, Shanghai (China). The in vivo PK properties of prodrug **ELQ-598** and its active metabolite **ELQ-596** were evaluated following po (10 mg/kg) and iv (0.3 mg/kg) administration to male CD1 mice. The mice were between 30 and 32 g and 6 and 8 weeks old at the start of the study. The vehicle used for PO dosing was PEG400, whereas a vehicle composed of 10% DMSO, 10% Kolliphor Solutol, and saline was used for iv dosing of **ELQ-598**. Animals had free access to food and water during the study. Following drug administration, the animals were manually restrained at the designated time points, and 100 μL of blood was withdrawn from the facial vein for semiserial sampling into heparinized tubes. Blood samples were placed on ice and centrifuged (2000*g*, 5 min, 4 °C) to obtain plasma. The plasma was mixed with 10% formic acid (FA) in water [plasma/10% FA (v/v = 10:1)]. Samples were taken at 15 and 30 min and 1, 2, 4, 8, 24, 48, 72, 96, and 120 h. At the outset of the study, 18 animals were randomly separated into two different groups (po and iv) of nine animals each. Blood samples were taken from three animals at each time point such that the same group of the animals was sampled at every fourth time point throughout the experiment for both po and iv arms. The plasma concentrations of **ELQ-598** and **ELQ-596** were analyzed by a validated LC–MS/MS method using a Siex Triple Quad 6500 instrument equipped with a Waters AQUITY HPLC column HSS T3 1.8 μm (2.1 × 50 mm). Separation was performed by reverse phase chromatography with the following conditions: mobile phase A: H_2_O–0.025% FA–1 mM NH_4_OAc and mobile phase B: MeOH–0.025% FA–1 mM NH_4_OAc with a gradient (%B) of 5% (0.2 min), 95% (0.6 min), 95% (1.40 min), 5% (1.41 min), and stop (1.80 min). Dichlofenac was used as an internal standard. The flow rate was 0.60 mL/min, and the column separation temperature was set to 60 °C. Under these conditions, the retention time was 1.31 min for **ELQ-598** and 1.20 min for **ELQ-596** with an LLOQ (lower limit of quantification) of 1 ng/mL. Concentration vs time profiles were analyzed using the Phoenix WinNonLin version 8.3.5.340 software as implemented by Certara (Princeton, NJ) to derive the PK values. The dose input of **ELQ-598** was 10 mg/kg for the po arm and 0.3 mg/kg for the iv arm. Based on MW and assuming that all **ELQ-598** was converted to **ELQ-596**, the estimated dose input of **ELQ-596** was 8.19 mg/kg for the po arm and 0.25 mg/kg for the iv arm. The AUC ratio for **ELQ-598** to **ELQ-596** and the absolute bioavailability (*F*) were calculated according to the following equations:
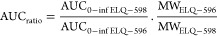

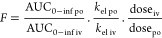


MW is the molecular weight. The terminal elimination rate constant *k*_el_ was estimated by linear least-squares regression analysis of the terminal log-linear portion of the plasma concentration–time curves by the software.

#### Homology Modeling and Docking

The crystal structure of *P. falciparum* cytochrome *bc*_1_ complex has not been resolved, and hence, homology modeling was performed to model the cytochrome *b* subunit. The protein sequence of the *P. falciparum* subunit b was obtained from the UniProt database,^37^ and a template search was initiated with BLAST using the PDB database. The bovine heart cytochrome *bc*_1_ complex subunit b (PDB ID 4D6T(38)) was identified as a template, and homology modeling was performed using the Molecular Operating Environment (MOE) software^39^ to generate 10 best models ranked based on their total potential energy. The best ranking model was chosen and subjected to further refinement with conjugate gradient minimization and 20 ns of molecular dynamics simulations to relieve any intramolecular steric constraints. All simulations were performed using AMBER charges and force field.^40^ The 3D structures of all the ELQ compounds were modeled using the builder module of MOE and optimized with AMBER charges and force field. Molecular docking was performed with the docking software^41^ with 30 poses of each ligand with complete receptor and ligand flexibility to the Q_i_ site of the cytochrome *b* unit. The docked poses were ranked with GOLDSCORE, and the best ranking pose was utilized for further analysis.

## Supporting Information Available

ORTEP diagram of **ELQ-331** in crystalline form (Figure S1); ORTEP diagrams of isoform **ELQ-598A** and **ELQ-598B** (Figure S2); ORTEP packing diagram showing the interaction between adjacent molecules in the prodrug **ELQ-331** crystal (Figure S3) and the prodrug **ELQ-598** crystal (Figure S4); and scatter plot of pIC_50_ (Figure S5) (PDF)
