# Supplementary material for: 3-Position Biaryl Endochin-like Quinolones with Enhanced Antimalarial Performance
Source: ACS Infect Dis. 2024 Jun 11;10(7):2419–42. doi: 10.1021/acsinfecdis.4c00140 (PMC11245370; doi:10.1021/acsinfecdis.4c00140)
Supplement: Supplementary file 1 — id4c00140_si_001.pdf [file id4c00140_si_001.pdf]

Supplemental Information to accompany:
ACS Infectious Diseases
Authors: Sovitj Pou et al,
Title: *3-Position Biaryl Endochin-Like Quinolones with Enhanced Antimalarial Performance*

1. Comparative crystal structure information for prodrugs ELQ-598 and ELQ-331.

Crystal structures of ELQ-331 and ELQ-598: The 3D crystal structures of antimalarial prodrugs, ELQ-331 and ELQ-598, were determined by x-ray diffraction analysis. In the crystal structure of ELQ-598 there are two symmetrically independent molecules with the same molecular structure, but with slightly different conformational parameters **(Figure S1)**. The central N1,C1-C9 fragments in both ELQ-598 molecules are planar inside of 0.0232 and 0.0314 Å. Similarly, the central N1, C1-C9 quinolone fragment of ELQ-331 is planar within 0.029 Å **(Figure S2)**. The torsion angles C3-C2-C12-C13 between the quinoline group and the innermost phenyl group attached to it for both ELQ-331 and ELQ-598 are similar with a value of 62.4(2)° and 62.9(2)°, respectively. For ELQ-331, the two terminal groups of the diaryl ether group are bent away from the central part of the molecule. Dihedral angles between the average planes of the N1, C1-C9, C12-C17 and C18-C23 fragments are 6.16(5)º and 71.75(7)º, respectively. For ELQ-598, the phenyl rings, C12-C17 and C18-C23, are twisted relative to each other by 36.77(9)° and 33.6(1)°. The dihedral angles between the average planes of the N1, C1-C9 fragment and the phenyl ring C12-C17 in both molecules are close, 65.59(4)° and 65.77(5)°. The atoms C12 and C12' of the phenyl rings directly bonded to the central part of the molecule are slightly out from the average plane of the central portion, 0.1067(22) and 0.1124(24) Å. The alkoxy carbonate promoiety groups are bent out and away from the central part of the molecule to minimize steric interaction with a torsion angle C25-O4-C3-C2 of 62.6 (2)° for **ELQ-331** and C25-O3-C3-C2 of 70.5 (2)° for **ELQ-598**. We include an overlay diagram in **Figure S3** with the structure of **ELQ-331** in bold while the structure of **ELQ-598** is shown with dashed lines. The image shows that the quinoline core nucleus remains a constant structural feature of both molecules while the rigid biaryl ring system of **ELQ-598** projects into space not occupied by the angular diphenylether side chain of **ELQ-331**.

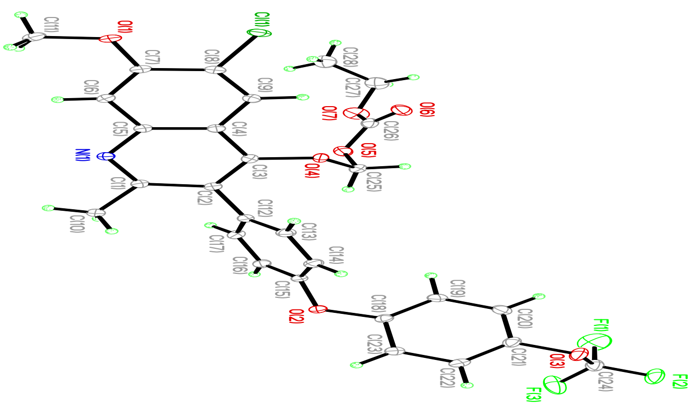

Figure S1. ORTEP diagram of ELQ-331 in crystalline form. Ellipsoids are drawn at the 30% probability level.

ELQ-598A ELQ-598B

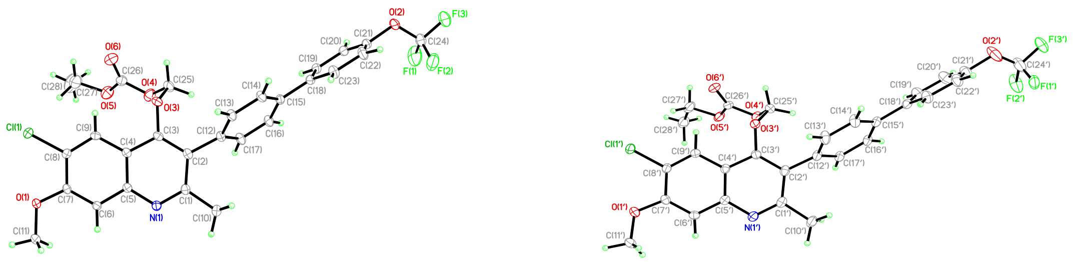

Figure S2. Two different isoforms of ELQ-598 existing in the crystalline state as described below. Shown are the ORTEP diagrams for isoform ELQ-598A (left panel) and ELQ-598B (right panel). Ellipsoids are drawn at the 30% probability level. The structures are described in greater detail in the text below.

Packing diagrams for the two drugs are shown below in **Figures S3** and **S4**. The ordered **ELQ-331** crystal is held together by an extensive array of π – π intermolecular interactions between the quinoline cores as well as the two aromatic rings of the diphenylether side chains. The shortest distance between the average planes of the quinoline rings fragments is 3.8912 Å. The hydrogen bonds as shown by the dotted lines between the C-H•••O interactions also

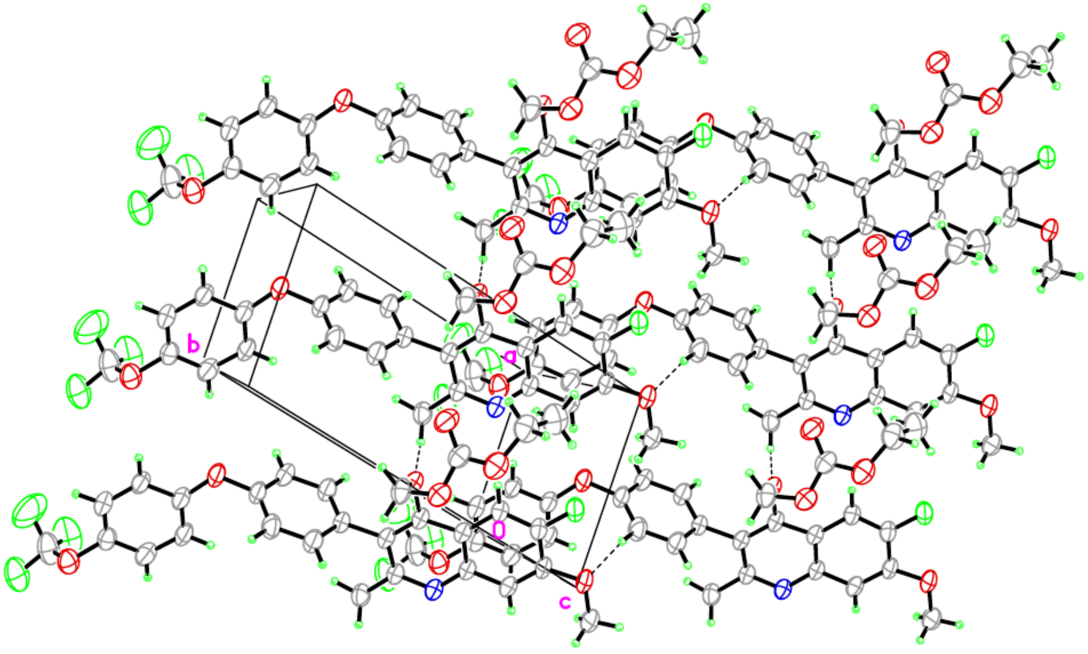

Figure S3. The ORTEP packing diagram showing the interaction between adjacent molecules in the prodrug ELQ-331 crystal.

strengthen the packing order for **ELQ-331**. In the crystal packing structure for **ELQ-598** we observe a similar display of intermolecular bonding forces holding the structure together in an ordered arrangement. Evident are π - π interactions between adjacent quinoline cores as well as the 3-position biaryl projection. The shortest distance between the average planes of the quinoline ring fragments is 3.4240 Å. We highlight critical hydrogen bonds as shown by the dotted lines between the C-H•••O interactions that add strength to the overall crystal lattice cohesion. The found bond distances in both crystal structures are comparable with the standard values of the corresponding bonds.

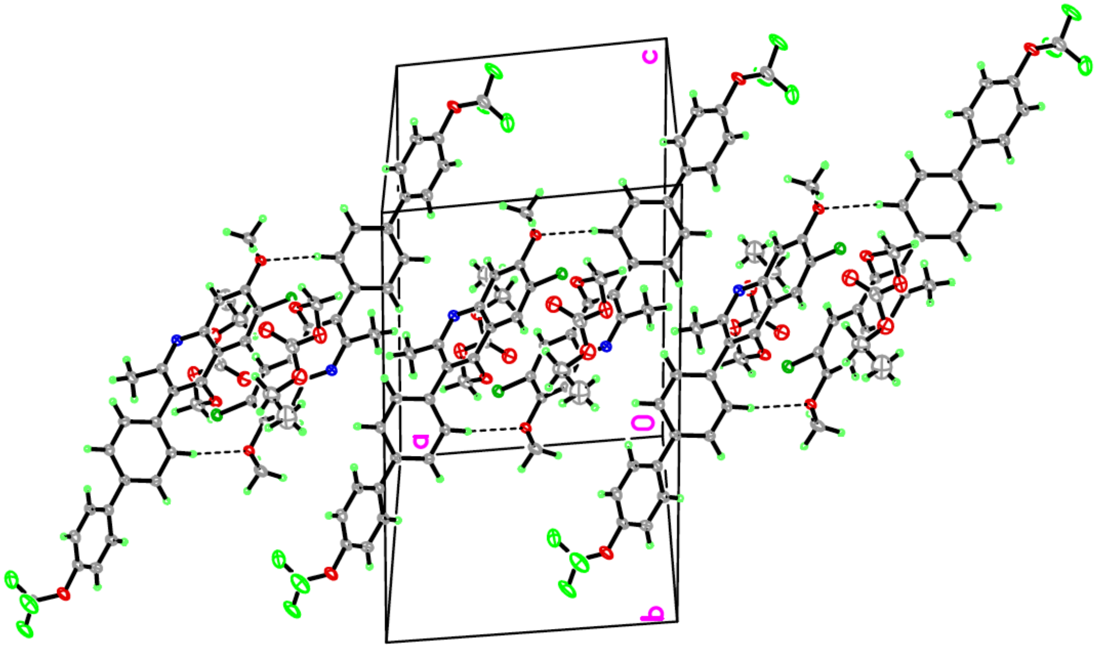

Figure S4. The ORTEP packing diagram showing the interaction between adjacent molecules in the prodrug ELQ-598 crystal.

X-Ray Crystallography Associated Methods and Analysis:

X-ray Crystallography. Diffraction intensities for **ELQ-598** and **ELQ-331** were collected at 173 K on a Bruker Apex2 CCD diffractometer using a *Incoatec* Cu *IμS* source, CuKα radiation, 1.54178 Å. Space group was determined based on intensity statistics. Absorption correction was applied by SADABS.[1] Structure was solved by direct methods and Fourier techniques and refined on F^2 using full matrix least-squares procedures. All non-H atoms were refined with anisotropic thermal parameters. H atoms were found on the residual density map and refined with isotropic thermal parameters. All calculations were performed by the Bruker SHELXL-2014/7 package.[2]

Crystallographic Data for **ELQ-598**: $C_{28}H_{23}ClF_3NO_6$, M = 561.92, 0.20 x 0.19 x 0.14 mm, T = 173(2) K, Triclinic, space group P-1, a = 11.3513(6) Å, b = 14.3260(8) Å, c = 16.8752(10) Å, α = 105.534(3)°,

β = 96.717(3)°, γ = 95.607(3)°, V = 2601.8(3) $Å^3$, Z = 4, Dc = 1.435 Mg/m^3, μ(Cu) = 1.885 mm^{-1}, F(000) = 1160, $2θ_{max}$ = 137.750°, 31034 reflections, 9184 independent reflections [R_{int} = 0.0364], R1 = 0.0426, wR2 = 0.1180 and GOF = 1.055 for 9184 reflections (914 parameters) with I>2σ(I), R1 = 0.0465, wR2 = 0.1217 and GOF = 1.068 for all reflections, max/min residual electron density +0.596/-0.457 $eÅ^{-3}$.

Crystallographic Data for **ELQ-331**: $C_{28}H_{23}ClF_3NO_7$, M = 577.92, 0.17 x 0.14 x 0.12 mm, T = 173(2) K, Triclinic, space group P-1, a = 8.2660(4) Å, b = 12.2303(6) Å, c = 13.8274(7) Å, α = 98.357(2)°, β = 106.128(2)°, γ = 98.365(3)°, V = 1302.97(11) $Å^3$, Z = 2, D_c = 1.473 Mg/m^3, μ(Cu) = 1.929 mm^{-1}, F(000) = 596, $2θ_{max}$ = 133.27°, 12072 reflections, 4581 independent reflections [R_{int} = 0.0437], R1 = 0.0479, wR2 = 0.1294 and GOF = 1.050 for 4581 reflections (453 parameters) with I>2σ(I), R1 = 0.0526, wR2 = 0.1345 and GOF = 1.050 for all reflections, max/min residual electron density +0.936/-0.511 $eÅ^{-3}$.

Structural Biology and Computer Docking of ELQ ligands into the Pf cytochrome bc_1 homology model.

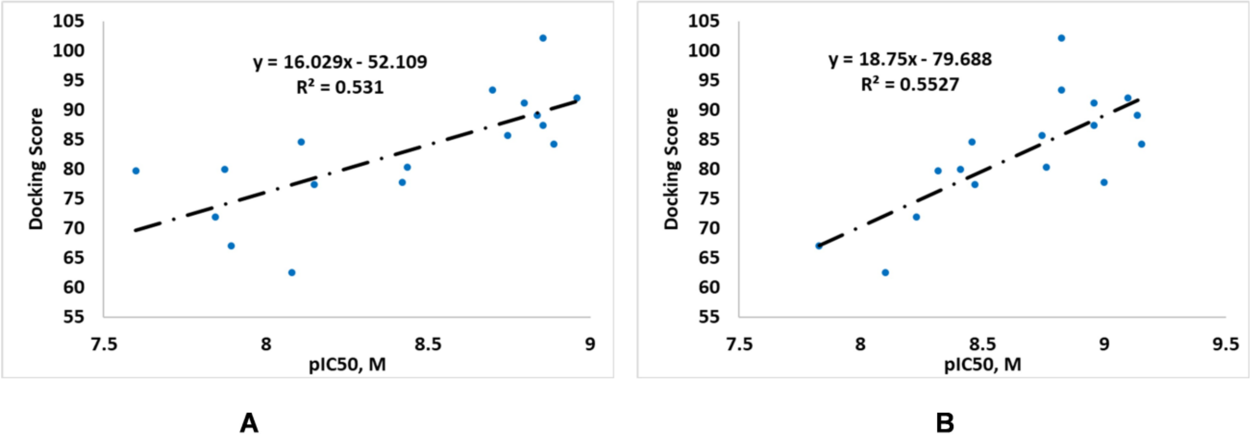

Fig S5. Scatter plot of pIC_{50} (in M) on X-axis and docking score on Y-axis shows positive correlation for docking scores vs efficacy in Dd2 (A) and D6 (B) parasites. Docking scores were derived from GOLD docking software with higher scores denoting better binding efficiency.

References

(1) Sheldrick, G. M. Bruker/Siemens Area Detector Absorption Correction Software Program. In *Bruker AXS*, 1998.

(2) Sheldrick, G. M. Crystal structure refinement with SHELXL. *Acta. Cryst.* **2015**, *C71*, 3-8.
